# Intra- and inter-network connectivity abnormalities associated with surgical outcomes in degenerative cervical myelopathy patients: a resting-state fMRI study

**DOI:** 10.3389/fneur.2024.1490763

**Published:** 2024-11-06

**Authors:** Yuqi Ge, Jiajun Song, Rui Zhao, Xing Guo, Xu Chu, Jiaming Zhou, Yuan Xue

**Affiliations:** ^1^Department of Orthopedic Surgery, Tianjin Medical University General Hospital, Tianjin, China; ^2^Department of Orthopedics, Xijing Hospital, Fourth Military Medical University, Xi'An, China; ^3^Department of Orthopedics, Cangzhou Central Hospital, Cangzhou, Hebei, China; ^4^Department of Shoulder and Elbow of Sports Medicine, Honghui Hospital, Xi'an Jiaotong University, Xi'An, China; ^5^School of Medical Imaging, Tianjin Medical University, Tianjin, China; ^6^Tianjin Key Laboratory of Spine and Spinal Cord, Tianjin Medical University General Hospital, Tianjin, China

**Keywords:** functional MRI, independent component analysis, network connectivity, degenerative cervical myelopathy, dynamic functional connectivity, support vector machine

## Abstract

Resting-state functional MRI (fMRI) has revealed functional changes at the cortical level in degenerative cervical myelopathy (DCM) patients. The aim of this study was to systematically integrate static and dynamic functional connectivity (FC) to unveil abnormalities of functional networks of DCM patients and to analyze the prognostic value of these abnormalities for patients using resting-state fMRI. In this study, we collected clinical data and fMRI data from 44 DCM patients and 39 healthy controls (HC). Independent component analysis (ICA) was performed to investigate the group differences of intra-network FC. Subsequently, both static and dynamic FC were calculated to investigate the inter-network FC alterations in DCM patients. k-means clustering was conducted to assess temporal properties for comparison between groups. Finally, the support vector machine (SVM) approach was performed to predict the prognosis of DCM patients based on static FC, dynamic FC, and fusion of these two metrics. Relative to HC, DCM patients exhibited lower intra-network FC and higher inter-network FC. DCM patients spent more time than HC in the state in which both patients and HC were characterized by strong inter-network FC. Both static and dynamic FC could successfully classify DCM patients with different surgical outcomes. The classification accuracy further improved after fusing the dynamic and static FC for model training. In conclusion, our findings provide valuable insights into the brain mechanisms underlying DCM neuropathology on the network level.

## Introduction

Degenerative cervical myelopathy (DCM) is a prevalent cause of non-traumatic spinal cord injuries and chronic spinal cord dysfunction among adults. This condition is characterized by the compression of the spinal cord resulting from degenerative changes in the cervical spine. DCM patients experience a range of neurological symptoms, such as pain, sensory abnormalities, gait disturbance, and limb dyscoordination (1, 2). Previous neuroimaging studies have explored structural, functional and metabolic adaptive changes at the cortical level in DCM patients (3–5). Mounting evidence suggests that the gradual deterioration of spinal cord dysfunction, which transmits signals to and from the brain, has a significant impact on brain morphology and functional activity (6–9). Predicting surgical outcomes is crucial for spine surgeons. Traditional cervical MRI's reliability in predicting patient prognosis is debatable. Therefore, simple, accurate, and non-invasive imaging biomarkers are needed to predict postoperative neurological recovery in patients.

Recent studies have shifted focus from regional alterations to brain network reorganization, aiming to elucidate the neuropathology of DCM and develop potential prognostic biomarkers. Functional connectivity (FC) analysis utilizing resting-state functional MRI (fMRI) data revealed predictable alterations across different stages of DCM progression (3). Previous studies have constructed static (i.e., calculating the Pearson correlation coefficients between time series), fine-grained brain functional networks based on anatomical templates, overlooking coarse-grained FC that provide the holistic interplay between various functional systems. Independent component analysis (ICA) is a data-driven method that can investigate coarse-grained inter-network FC by blindly separating neural signals from multiple brain systems (10, 11). Furthermore, the brain is a complex dynamic system, and the strength and variability of FC can vary rapidly at timescales of seconds to minutes (12). To investigate the dynamic architecture of the brain networks, the dynamic FC (dFC) analysis using the sliding window approach has provided valuable insights into the temporal dynamic changes in FC (13). However, this method has limitations, including the use of arbitrarily chosen fixed-length windows and the disregard of transient FC modes (14). To overcome these limitations, novel approaches such as dynamical conditional correlation (DCC) and flexible least squares (FLS) algorithms have been proposed and shown to outperform the traditional sliding window approach (14–16). Prior investigations exclusively conducted univariate analysis to merely compare the amplitude of specific FC between patients and healthy controls (HC). In contrast, the multivariate approach offers an unmatched ability to detect distinctions in the spatial architecture of network modifications and reorganization between patients and HC. Multivariate approaches assess the intricate interactions among numerous variables, thereby facilitating accurate predictions (17).

Therefore, we investigated the coarse-grained FC alterations between DCM patients and HC utilizing ICA approach and calculated dFC to investigate the temporal variability of coarse-grained FC. Furthermore, we conducted a comparative assessment of the predictive efficacy of static FC (sFC), dFC, and their fusion in classifying DCM patients with favorable and poor prognoses.

## Materials and methods

### Subjects

Ethical approval for this retrospective study was granted by the institutional local review board. The inclusion criteria for DCM patients was as follows: (a) clear MRI evidence of cord compression on the cervical spine; (b) explicit clinical manifestations of myelopathy (sensorimotor extremity deficits, bladder/bowel dysfunction, gait disturbance, etc.); (c) patients agreed to undergo decompression surgery; (d) no history of cervical spinal surgery; (e) ability to complete fMRI studies; (f) no stenosis of extracranial vertebral artery and the carotid artery after Doppler ultrasound examination; (g) no clinical evidence or history of other neurological, psychiatric, ocular disease, or systemic disease, including hypertension and diabetes after consulting specialists in neurology, cardiology, and ophthalmology; (h) no history of alcohol and substance abuse. HC of similar age, gender, and education were also recruited through advertisements with the following inclusion criteria:(a) no evidence of spinal compression; (b) no other spinal or brain neurological disorders, or systemic disease; (d) ability to complete rs-fMRI studies. All participants gave written informed consent. Finally, 44 right-hand DCM patients and 39 HC were recruited continuously in 2020–2022 at Tianjin Medical University General Hospital.

### Clinical evaluation

Before fMRI scanning, a senior orthopedic surgeon conducted a thorough evaluation of DCM patients using the Japanese Orthopedic Association (JOA) scale (normally 3 days before decompression surgery). The postoperative JOA score was obtained at one year postoperatively. JOA recovery rate was calculated using the following formula.


$$\begin{array}{l} JOA\;recovery\;rate=\\ \frac{Postoperative\;JOA\;score-Preoperative\;JOA\;score}{17-Preoperative\;JOA\;score} \end{array}$$

The duration of the symptoms was also noted when the history was taken.

### MRI data acquisition and preprocessing

**Figure 1:**
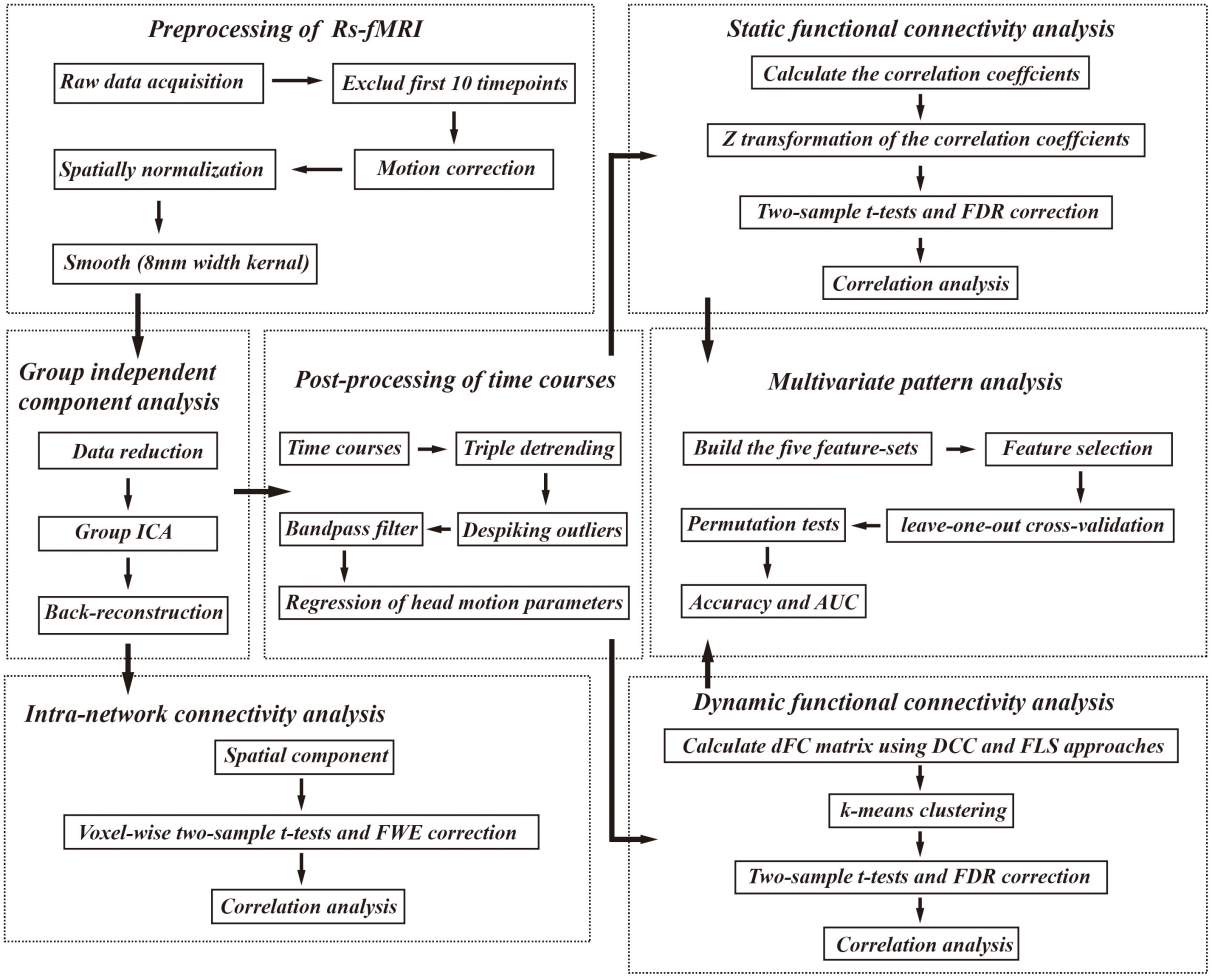
The analysis pipeline of our study. Rs-fMRI, resting state fMRI; FWE, familywise error; FDR, false discovery rate; AUC, area under the curve, DCC, dynamical conditional correlation; FLS, flexible least squares.

MRI scanning was performed on HC and preoperative patients. Data were acquired using a MAGNETOM Prisma 3 T MR scanner (Siemens, Erlangen, Germany) with a 64-channel phase-array head–neck coil. All participants used spongy pads to support their heads to minimize head movement during the scan. Participants were also asked to close their eyes and remain awake while avoiding specific and strong thoughts. Functional images were collected using a gradient echo-planar pulse imaging sequence with the following parameters: echo time (TE) = 30 ms; repetition time (TR) = 800 ms, field of view (FOV) = 222 × 222 mm; matrix = 74 × 74; in-plane resolution = 3 × 3 mm; flip angle (FA) = 54°; slice thickness = 3 mm; gap = 0 mm; number of slices = 48; slice orientation = transversal; bandwidth = 1,690 Hz/pixel, parallel acquisition technique mode; slice acceleration factor = 4; phase encoding acceleration factor = 2. Four hundred fifty images were taken in 6 min. A high-resolution 3D T1 structural image (2 inversion contrast magnetization prepared rapid gradient echo sequence, MP2RAGE) was also acquired using the following parameters: TR/TE = 4,000 ms/3.41 ms, inversion times (TI1/TI2) =700 ms/2,110 ms, FA1/FA2 = 4 degree/5 degree, matrix = 256 × 240, FOV = 256mm × 240mm, number of slices = 192, in-plane resolution = 1 mm × 1 mm, slice thickness = 1 mm, slice orientation = sagittal, total duration is 6 min, 42 s. fMRI data were preprocessed using the Data Processing Assistant for rs-fMRI (http://www.restfmri.net/forum/DPARSF) toolbox. The first 10 volumes of each function scan were excluded for the adaption of participants and magnetization stability. Motion correction was performed to eliminate the effects of head movement. The functional images were co-registered to structural images and spatially normalized to the Montreal Neurological Institute template, each voxel was resampled to 3 × 3 × 3 mm^3^. Finally, resampled images were smoothed with an 8-mm full-width-half-maximum isotropic Gaussian kernel. Figure 1 illustrates the analysis pipeline of our current study.

### Group independent component analysis

All preprocessed images were analyzed using the GIFT software (mialab.mrn.org/software/gift/). The ICA analysis involved three main steps: (1) data reduction, (2) group ICA, and (3) back-reconstruction. In this study, 17 independent components (IC) were estimated using the minimum description length criteria, and principal component analysis was applied for data dimensionality reduction. The infomax algorithm was employed for group ICA analysis, and the ICASSO method was utilized with 100 repetitions to ensure data repeatability (18). Finally, the spatial map and time course specific to each subject were reverse reconstructed using spatial-temporal regression. A binary mask was obtained for each resting-state network (RSN) using one-sample *t*-tests for DCM patients and HC respectively, with correction for familywise error (FWE) at voxel-level *P* < 0.001 and cluster-level *P* < 0.05. Subsequently, a binary mask was obtained by intersecting these two masks from DCM patients and HC for further visualization. Next, within the intersection of these two masks, spatial maps of each RSN were gathered across all the participants by one-sample *t*-tests corrected by FEW correction (voxel-level *P* < 0.001, cluster-level *P* < 0.05).

To ensure result validity, we followed the criteria outlined in a prior study (13). We confirmed that the peak activation coordinates of spatial maps primarily resided in the gray matter, showing low overlap with known vascular, ventricular, motion, and susceptibility artifacts and the time course of the RSN was dominated by low-frequency fluctuations. Furthermore, the dice similarity coefficient (DSC) was calculated between the binary masks of the spatial maps and the templates provided in the GIFT software [the RSN template (19) and Neuromark template (20)]. The DSC was calculated using the following formula.


$$\begin{array}{l} DSC_{A,B}=\frac{2\times \left(A\cap B\right)}{A+B} \end{array}$$

where A and B represent the compared masks, AnB represents the number of common voxels between A and B, and A + B represents the total number of voxels for A and B.

Finally, 11 IC were selected for further analyses: medial visual network (MVN), lateral visual network (LVN), cerebellar network (CBN), anterior default mode network (aDMN), posterior default mode network (pDMN), attention network (AN), auditory network (AUN), left frontoparietal network (LFPN), right frontoparietal network (RFPN) and sensory network (SN) and motor network (MN).

### Intra-network connectivity analysis

To investigate the intra-network connectivity, the spatial component for above-mentioned brain networks was compared between DCM patients and HC. To ensure the analysis focused on “intra-network FC”, we applied a network specific-mask (the binary mask of selected RSN in the previous analysis) when performing two-sample *t*-tests with age, gender, and education years as covariates, the results were corrected using FWE correction (voxel-level *P* < 0.001, cluster-level *P* < 0.05).

### Internetwork connectivity analysis

To explore the inter-network connectivity, the time courses of above mentioned 11 components were extracted and performed post-processing steps, including triple detrending (linear, cubic, quadratic), despiking detected outliers by 3dDespike algorithm, low-pass filtering with a high cutoff frequency of 0.08 Hz, and linear regression of the Friston 24 head motion parameters. Continuing, both sFC and dFC between these IC were calculated.

For sFC, pairwise correlations of the time series were calculated and then Fisher-Z transformed to obtain the FC matrix (11 × 11). For dFC, we simultaneously use DCC and FLS approaches. DCC method (https://github.com/canlab/Lindquist_Dynamic_Correlation) involves fitting a generalized autoregressive conditional heteroskedasticity model to all time series and estimating time-varying correlations from the standardized residuals (21). For FLS method, we used DynamicBC toolbox (16), which employs a distribution-free time-varying parameter regression strategy. FLS algorithm assigns two types of residual errors to each coefficient sequence estimate: squared residual measurement errors and squared residual dynamic errors. Pairwise dFC values were obtained for each participant, resulting in a dFC matrix of 440 (time points) × 121 (connections). These matrices were Fisher-Z transformed. Two metrics were computed for each pairwise dFC: the temporal mean value ($\bar{FC}$) and the temporal variability (*FC*_δ_) (e.g., standard deviation) across time.

To identify reoccurring dFC patterns (states), we applied a k-means clustering algorithm with the cityblock distance. Silhouette index and Calinski-Harabasz index were used to determine the optimal number of clusters. Temporal properties of dFC states were analyzed using three variables: (1) fraction time, representing the percentage of the total windows belonging to one state; (2) mean dwell time, indicating the number of consecutive windows belonging to one state; and (3) number of transitions, representing the sum of time points in which the state changed.

Finally, two-sample *t*-tests were performed to reveal the differences in sFC matrix, $\bar{FC}$, *FC*_δ_, fraction time, mean dwell time, and number of transitions with age, gender, and education years as covariates between DCM patients and HC and corrected using false discovery rate (FDR) correction (P <0.05).

### Correlation analysis

To investigate the potential association between brain network abnormalities and clinical assessment, Pearson correlation coefficients were calculated. For intra-network FC, correlations were calculated between the mean values within abnormal brain regions and clinical assessments. For inter-network FC, correlations were calculated between sFC, $\bar{FC}$, *FC*_δ_ of dFC, temporal properties of dFC (fraction time, mean dwell time, and number of transitions) and clinical assessments.

### Multivariate pattern analysis (MVPA)

To assess the predictive value of sFC and dFC for the prognostic prediction in DCM patients, MVPA was performed via support vector machine, using MVPANI toolbox (http://funi.tmu.edu.cn) with sigmoid kernel and default parameters to classify DCM patients with good recovery and poor recovery. We used a cut-off value of 75% (the median of the data in this study) of the JOA recovery rate for defining good and poor prognosis. This categorical variable was used as the outcome for the development of machine learning classifiers. The rationale for converting this continuous variable into a binary variable is threefold: (1) considering our limited sample size, binary variables often have a smaller range of values, which can make the model simpler; (2) converting continuous variables into binary variables can effectively deal with outliers; (3) continuous variables can be affected by measurement errors or noise. Discretizing continuous variables into binary variables helps reduce the impact of noise in the data, improving the stability and robustness of the model.

In the current study, five feature-sets were used for developing model: sFC-based feature-sets, dFC (FLS)-based feature-sets, dFC (DCC)-based feature-sets, fusion of sFC and dFC (FLS) feature-sets, and fusion of sFC and dFC (DCC) feature-sets. As the FC matrix is symmetric, only the upper triangle elements were used. For the sFC-based feature-sets, the vector with 55 features was extracted from the sFC matrix of each participant. For the dFC-based feature-sets, the vector with 110 features was constructed based on $\bar{FC}$ and *FC*_δ_ of dFC matrix for each subject along with the five temporal properties of dFC (e.g., number of transitions, fraction time, and dwell time of each state), resulting in 115 features for the feature-sets. For the fusion of sFC and dFC feature-sets, 55 features of sFC-based predictive model and 115 features of dFC-based predictive model were fused to generate a large vector with 170 features.

Classification accuracies were assessed by the leave-one-out cross-validation procedure for each feature-set respectively to overcome the loss of generalization due to the relatively small sample size in this study. Briefly, one of the available data points is retained and the model is trained using the rest of the data. Before model training, to remove redundant features and avoid overfitting of the model, we selected the least absolute shrinkage and selection operator as the method for feature selection. Subsequently, the model was trained based on the selected features and tested on the held-out data point. This process was repeated until all data points were retained once as a test sample. The corresponding *P*-value for the classification of good prognosis and poor prognosis was calculated from the null distribution obtained from 1,000 random permutation tests by randomly shuffling the labels of samples. The *P*-values were calculated as a proportion of the number of permutations generated that were greater than or equal to actual classification accuracy and *P* < 0.05 with FDR correction for multiple comparisons was considered statistically significant. The receiver operating characteristic (ROC) curve and the corresponding area under the curve (AUC) of each model were also calculated.

### Validation analysis

To further investigate the potential influence of head motion on our result, we conducted validation analysis and compared the head motion between DCM patients and HC. Framewise displacement values were calculated using 3 robust methods including Jenkinson method, Power method, and VanDijk method. Moreover, to further test the stability of our results, we set the number of IC in ICA to 12 and 22 respectively and performed similar sFC analysis for internal validation. Furthermore, in MVPANI, we also applied other predictive models, multiple kernels of SVM and adjusted the tunable parameters of the SVM with the sigmoid kernel to find the optimal model for the study.

## Results

### Demographic data and clinical assessment

Forty four DCM patients and 39 HC were enrolled in this study. The demographic data and clinical assessments of all participants are summarized in Table 1. No significant inter-group differences in age, gender, or education years (*P* < 0.05) were observed.

**Table 1:** Demographic data and clinical assessments of the current study.

**Characteristics**	**DCM (*n* = 44)**	**HC (*n* = 39)**	***p* value**
Age (years)	54.0 ± 10.7	53.7 ± 8.3	0.88
Gender (F/M)	22/22	19/20	0.91
Education (years)	11.2 ± 2.7	11.1 ± 3.3	0.85
JOA	11.4 ± 2.1		
JOA recovery rate	0.74 ± 0.16		
Disease's duration (years)	4.0 ± 2.1		

DCM, degenerative cervical myelopathy; HC, healthy controls; JOA, Japanese Orthopedic Association.

### Intra-network connectivity analysis

**Figure 2:**
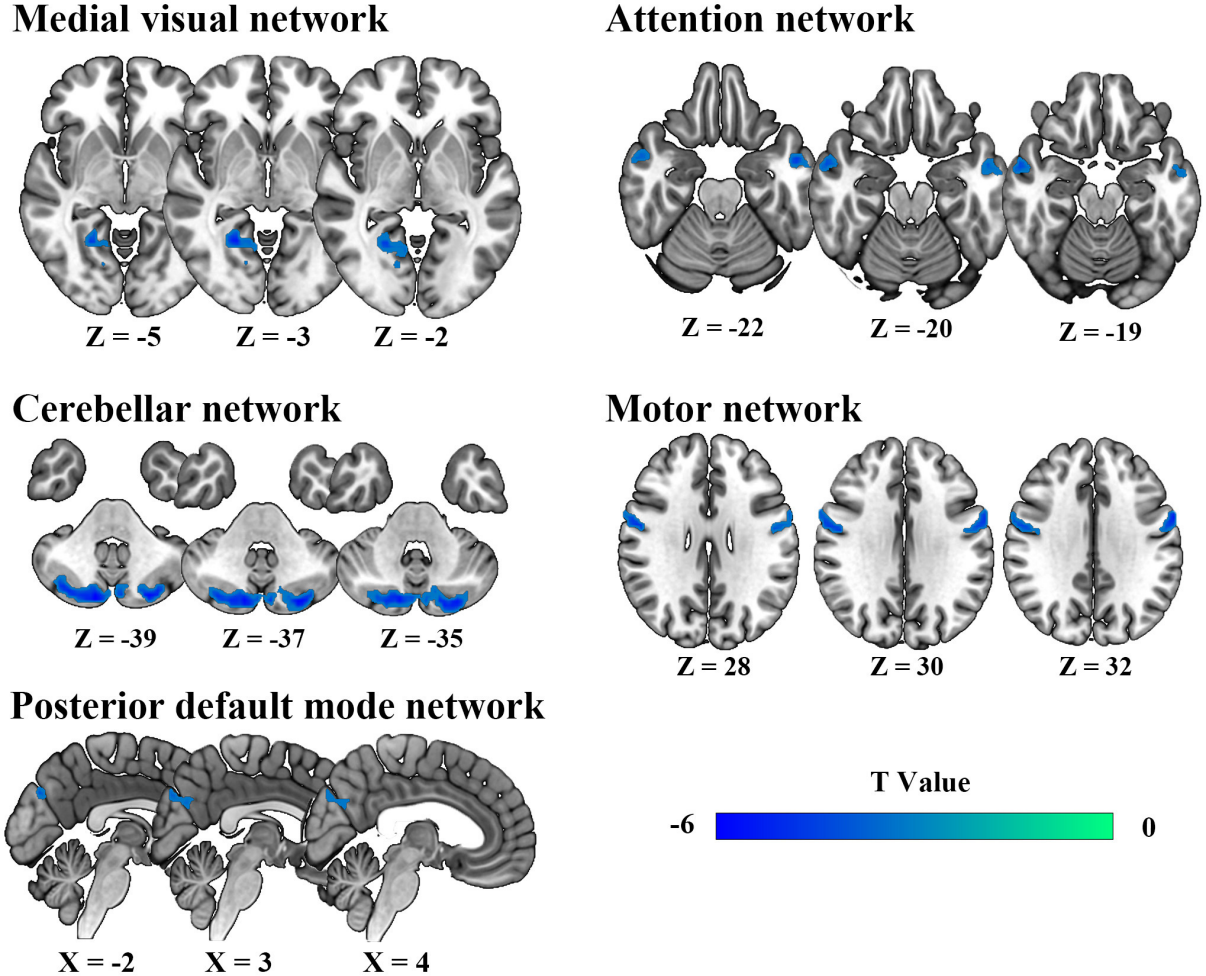
Abnormal intra-network static functional connectivity in degenerative cervical myelopathy patients compared to healthy participants.

Spatial maps of all 11 IC defined by group ICA and detailed information can be found in Supplementary Figure S1, Supplementary Table S1. The DSC between our spatial maps and RSN template obtained in the previous study were all above 0.52 (Supplementary Table S2), indicating that the obtained spatial maps in the study were robust. Compared to HC, DCM patients exhibited significantly lower intra-network FC between the right lingual gyrus and MVN, between bilateral cerebellum posterior lobe and CBN, between bilateral precuneus and pDMN, between left middle temporal gyrus, right inferior temporal gyrus, and AN, between bilateral precentral gyrus and MN (Table 2, Figure 2).

**Table 2:** Abnormal functional connectivity in each RSN between DCM patients and HC.

**RSN**	**Regions**	**BA**	**Voxels**	***T*-value**	**Peak MNI**		
					**x**	**y**	**z**
**DCM**<**HC**							
MVN	R lingual gyrus	18.19	84	−5.70	21	−54	−3
CBN	Bi cerebellum posterior lobe	—	333	−5.59	12	−84	−42
pDMN	Bi precuneus	23	58	−4.56	−3	−78	27
AN	L middle temporal gyrus	20	56	−4.90	−51	−6	−30
	L middle temporal gyrus	21	53	−5.90	−57	−9	−24
	R inferior temporal gyrus	20.21	35	−4.39	57	−6	−24
MN	L precentral gyrus	4.6	114	−5.88	−60	3	33
	R precentral gyrus	4.6	65	−5.15	54	−3	27

DCM, degenerative cervical myelopathy; HC, healthy controls; IC, independent component; MNI, Montreal Neurologic Institute; BA, Brodmann area; RSN, resting-state network; MVN, medial visual network; CBN, cerebellar network; pDMN, posterior default mode network; AN, attention network; MN, motor network; R, right; L, left; Bi, bilateral.

### Inter-network connectivity analysis

**Figure 3:**
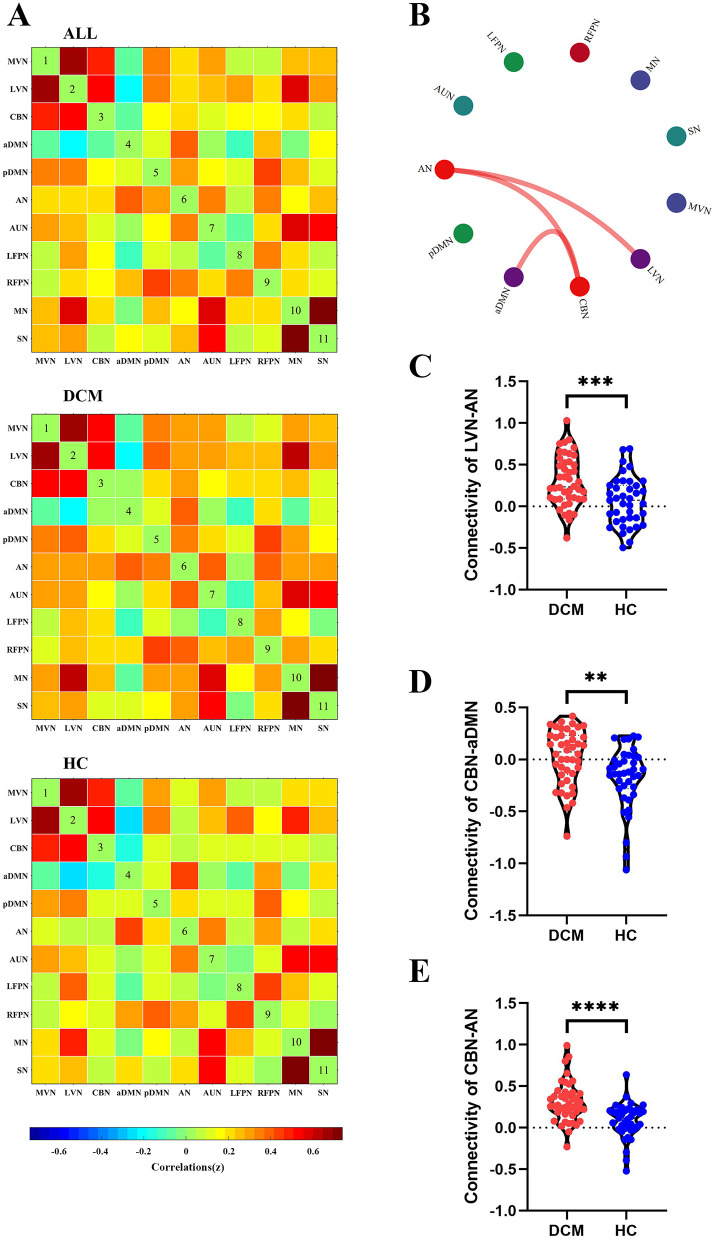
Abnormal inter-network static functional connectivity (sFC) in degenerative cervical myelopathy (DCM) patients compared to healthy controls (HC). **(A)** The heat map for averaged inter-network sFC pattern of all participants (upper panel), DCM patients (middle panel), and HC (lower panel). **(B)** The abnormal inter-network sFC between DCM patients and HC. The red lines indicated higher sFC in DCM patients. Abnormal inter-network sFC between lateral visual network (LVN) and attention network (AN) **(C)**, between cerebellar network (CBN) and anterior default mode network (aDMN) **(D)**, between CBN and AN **(E)**. MVN, medial visual network; pDMN, posterior default mode network; AUN, auditory network; LFPN, left frontoparietal network; RFPN, right frontoparietal network; SN, sensory network; MN, motor network. **In scatter plots means *p* < 0.01 (uncorrected), ^***^p < 0.001 (uncorrected), ^****^p < 0.0001 (uncorrected).

For sFC, the averaged sFC matrix for DCM patients, HC, and all participants were illustrated in Figure 3A. Compared with HC, DCM patients exhibited significantly higher sFC between AN and LVN (*P* < 0.001), between aDMN and CBN (*P* = 0.002), between AN and CBN (*P* < 0.001) (Figures 3B–E).

**Figure 4:**
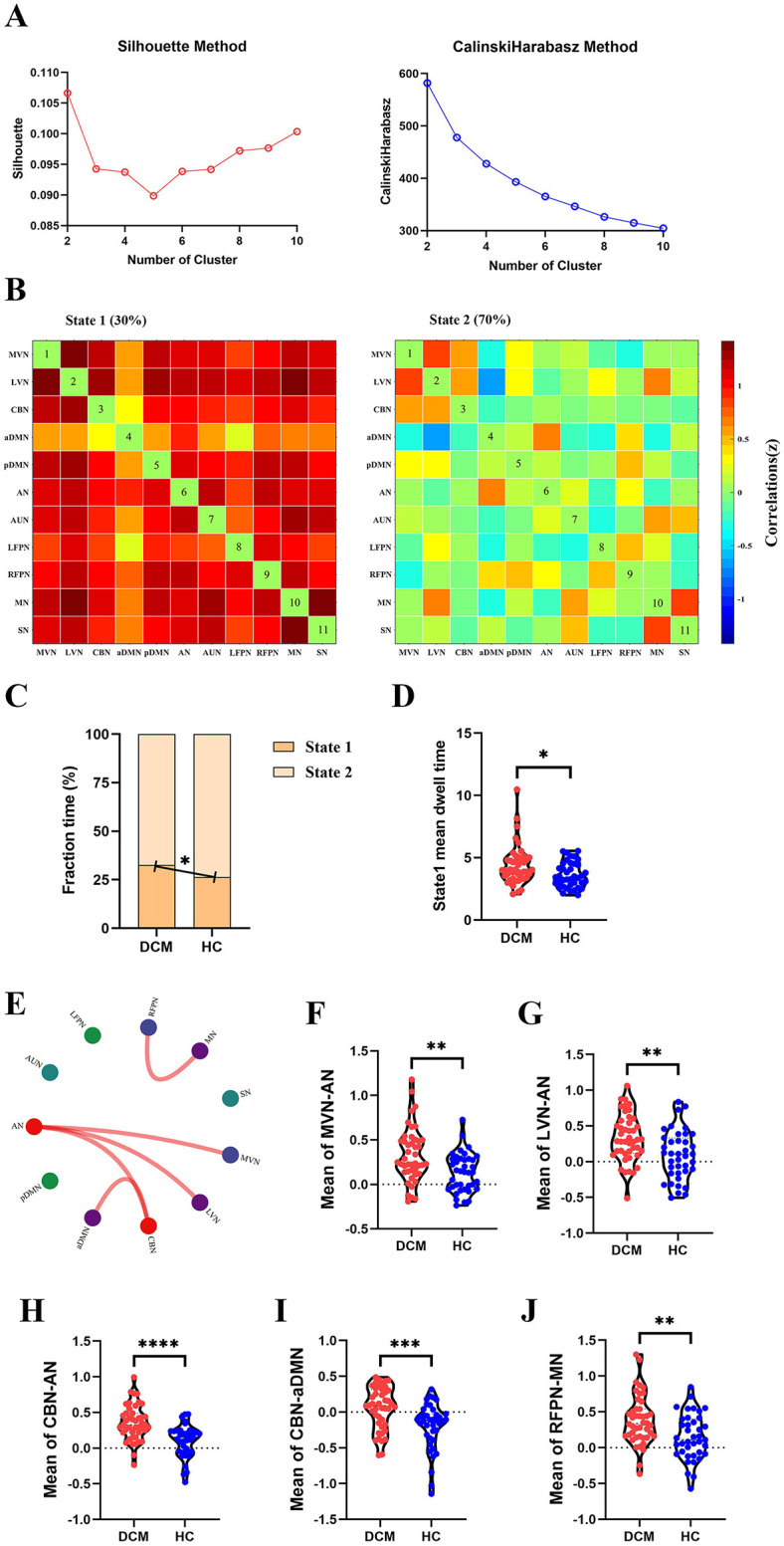
Abnormal dynamic functional connectivity (dFC) (dynamical conditional correlation approach) in degenerative cervical myelopathy (DCM) patients compared to healthy controls (HC). **(A)** The results of Silhouette index and Calinski-Harabasz index analyses, the optimal number of clusters was 2. **(B)** The median pattern of dFC for two types of states identified by k-means clustering analyses. The differences in fraction time **(C)** and mean dwell time **(D)** between DCM patients and HC. **(E)** The abnormal strength of dFC in DCM patients compared to HC. The red lines indicated higher strength of dFC in DCM patients. Inter-group differences in strength of dFC between medial visual network (MVN) and attention network (AN) **(F)**, between lateral visual network (LVN) and AN **(G)**, between cerebellar network (CBN) and AN **(H)**, between CBN and anterior default mode network (aDMN) **(I)**, between right frontoparietal network (RFPN) and motor network (MN) **(J)**. pDMN, posterior default mode network; AUN, auditory network; LFPN, left frontoparietal network; SN, sensory network. ^*^p < In scatter plots means *p* < 0.05 (uncorrected), ^**^p < 0.01 (uncorrected), ^***^p < 0.001 (uncorrected), ^****^p < 0.0001 (uncorrected).

**Figure 5:**
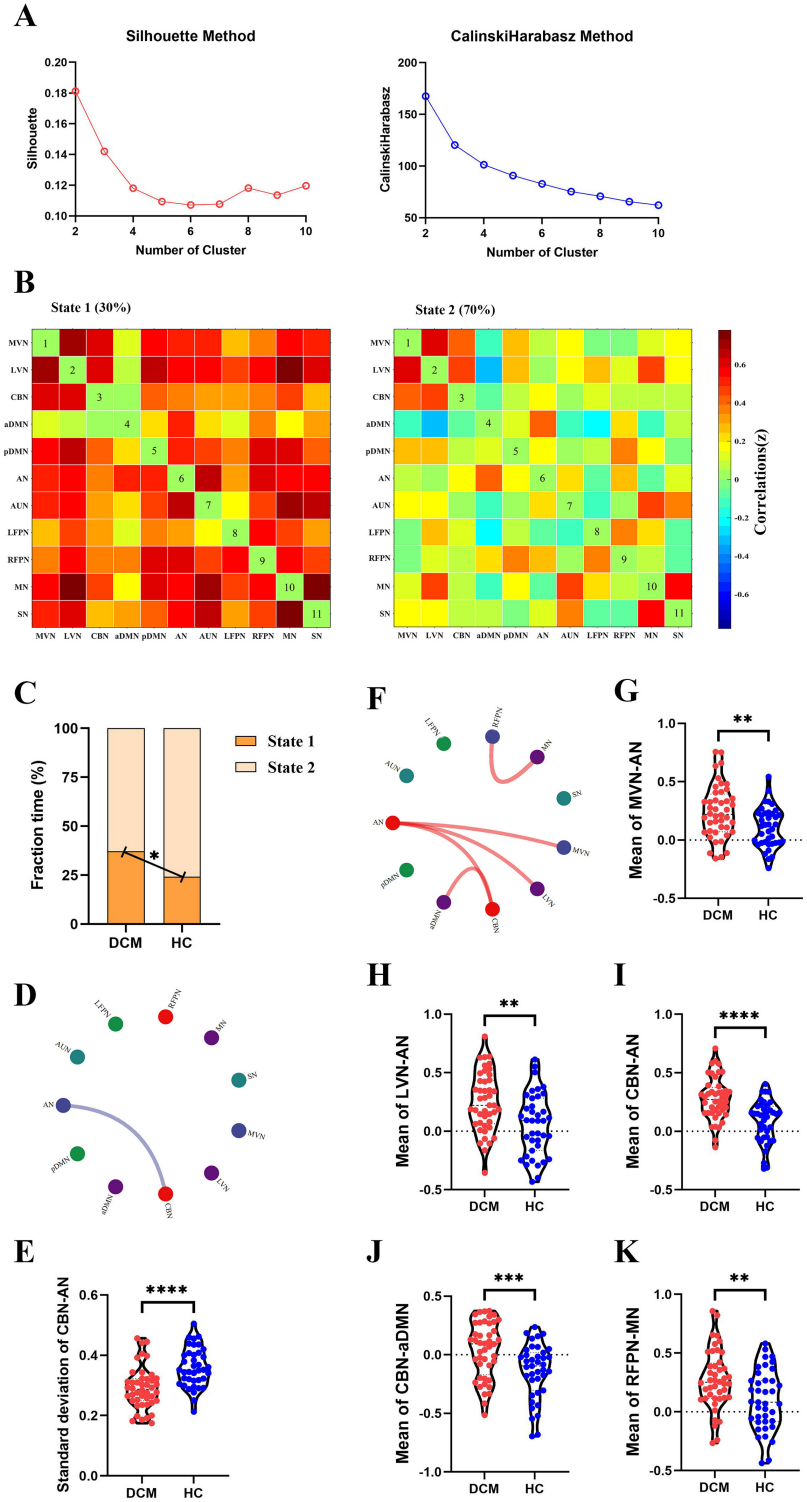
Abnormal dynamic functional connectivity (dFC) (flexible least squares approach) in degenerative cervical myelopathy (DCM) patients compared to healthy controls (HC). **(A)** The results of Silhouette index and Calinski-Harabasz index analyses, the optimal number of clusters was 2. **(B)** The median pattern of dFC for two types of states identified by k-means clustering analyses. **(C)** The differences in fraction time for each state between DCM patients and HC. The abnormal variability of dFC between cerebellar network (CBN) and attention network (AN) in DCM patients compared to HC, the blue lines indicated lower variability of dFC in DCM patients **(D, E)**. **(F)** The abnormal strength of dFC in DCM patients compared to HC. The red lines indicated higher strength of dFC in DCM patients. Inter-group differences in strength of dFC between medial visual network (MVN) and AN **(G)**, between lateral visual network (LVN) and AN **(H)**, between CBN and AN **(I)**, between CBN and anterior default mode network (aDMN) **(J)**, between right frontoparietal network (RFPN) and motor network (MN) **(K)**. pDMN, posterior default mode network; AUN, auditory network; LFPN, left frontoparietal network; SN, sensory network. **In scatter plots means *p* < 0.01 (uncorrected), ^***^p < 0.001 (uncorrected), ^****^p < 0.0001 (uncorrected).

For dFC, two approaches obtained partial overlap results. Firstly, the optimal number of clusters obtained by the Silhouette method and Calinski-Harabasz method was all two (Figure 4A, Figure 5A). Namely, a less frequent and relatively strongly connected state 1, and a more frequent and relatively sparsely connected state 2 (Figure 4B, Figure 5B). In addition, between-group comparison showed that state 1 was more frequent in DCM patients than HC (*P* = 0.01, Figure 4C; *P* = 0.02, Figure 5C); while the opposite pattern was observed for state 2 that was less frequent in DCM patients (*P* = 0.01, Figure 4C; *P* = 0.02, Figure 5C). Finally, compared with HC, DCM patients exhibited significantly higher level of $\bar{FC}$ of dFC between MVN and AN (*P* = 0.001), between LVN and AN (*P* = 0.001), between CBN and AN (*P* < 0.001), between CBN and aDMN (*P* < 0.001), between RFPN and MN (*P* = 0.002) (Figures 4E–J, 5F–K). Furthermore, in DCC method, the dwell time of state 1 was also higher in DCM patients (*P* = 0.01) (Figure 4D). In FLS method, DCM patients exhibited a significantly lower level of dFC variability between CBN and AN (*P* < 0.001) (Figures 5D, E).

### Correlation analysis

**Figure 6:**
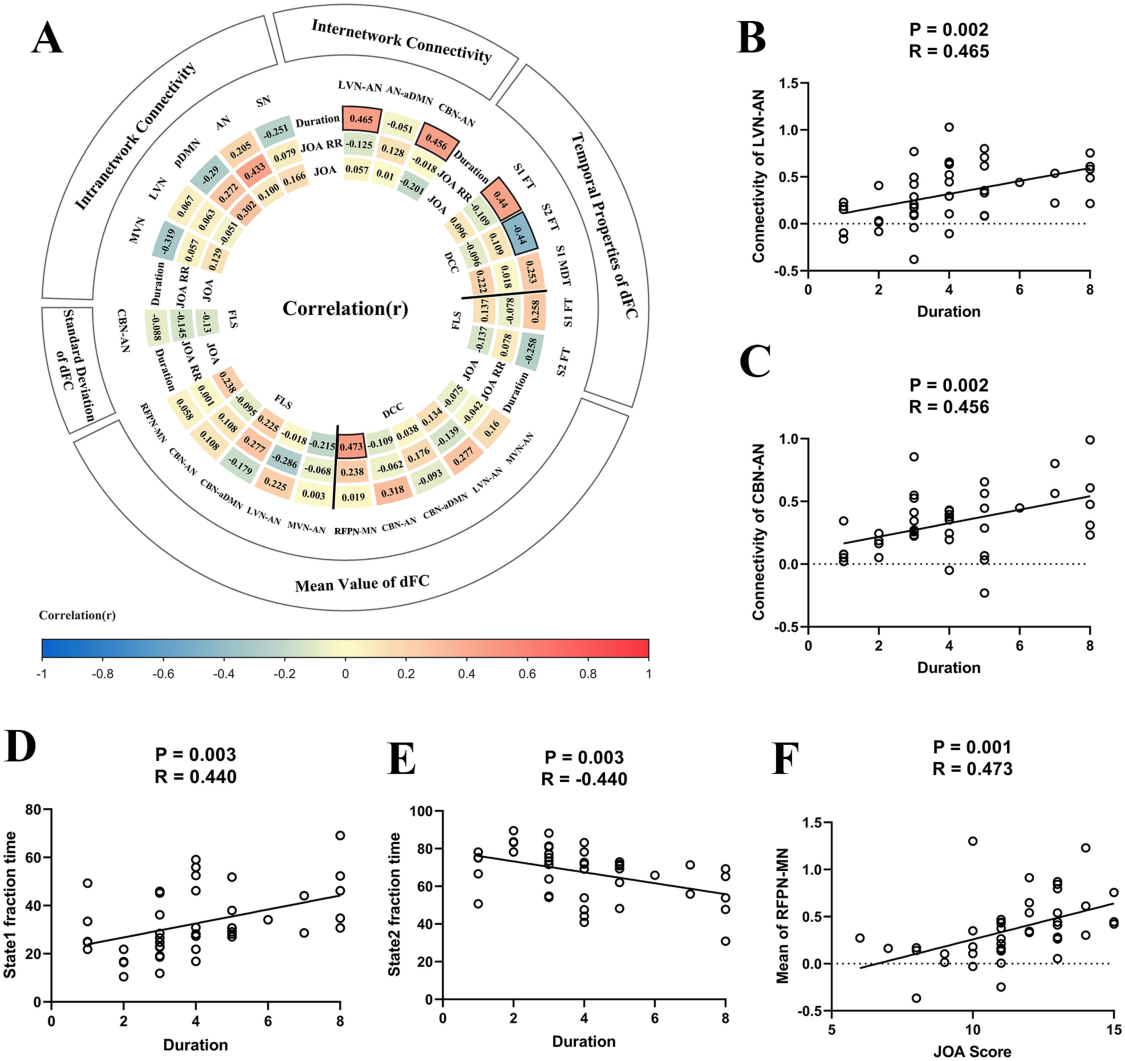
The results of correlation analysis. **(A)** The heat map for correlation coefficients between brain functional alterations and clinical assessments. Panel **(B)** The scatter plot for the association between static functional connectivity (sFC) [i.e., between lateral visual network (LVN) and attention network (AN)] and duration of the symptoms. **(C)** The scatter plot for association between sFC (i.e., between cerebellar network (CBN) and AN) and duration of the symptoms. **(D)** The fraction time of state 1 correlated with duration of the symptoms. **(E)** The fraction time of state 2 correlated with duration of the symptoms. **(F)** The strength of dFC between right frontoparietal network (RFPN) and motor network (MN) correlated with Japanese Orthopedic Association (JOA) score. DCC, dynamical conditional correlation; FLS, flexible least squares; S1, state1; S2, state2; FT, fraction time; MDT, mean dwell time; JOA RR, JOA recovery rate; MVN, medial visual network; aDMN, anterior default mode network; pDMN, posterior default mode network; AUN, auditory network; LFPN, left frontoparietal network; SN, sensory network.

Figure 6A illustrates the heat map for correlation coefficients between all brain abnormalities observed in our study and clinical assessments. For inter-network sFC, the abnormal sFC between LVN and AN correlated with duration of the symptoms (*P* = 0.002, R = 0.465, Figure 6B), the abnormal sFC between CBN and AN correlated with duration of the symptoms (*P* = 0.002, R = 0.456, Figure 6C). For dFC analysis, the fraction time of state 1 correlated with duration of the symptoms (*P* = 0.003, R = 0.440, Figure 6D), the fraction time of state 2 correlated with duration of the symptoms (*P* = 0.003, R = −0.440, Figure 6E), and the abnormal $\bar{FC}$ of dFC between RFPN and MN correlated with JOA score (*P* = 0.001, R = 0.473, Figure 6F). Moreover, the abnormal intra-network connectivity within the AN was correlated with the postoperative JOA recovery rate (*P* = 0.003, R = 0.433). Unfortunately, this result could not pass the FDR correction. The results of *P*-value before FDR correction can be found in Supplementary Figure S2.

### MVPA: good prognosis vs. poor prognosis classification

**Figure 7:**
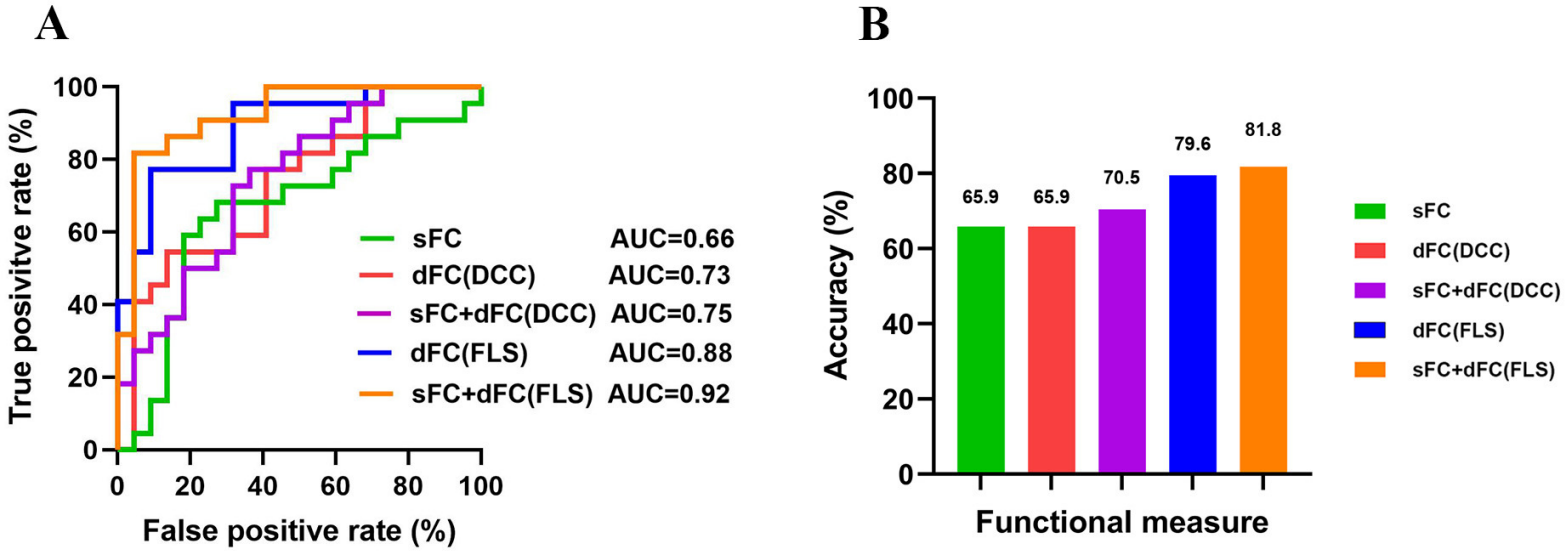
The results of multivariate pattern analysis. **(A)** The receiver operating characteristic (ROC) curve for classification models based on static functional connectivity (sFC), dynamic functional connectivity (dFC), and fusion of both metrics (sFC+dFC). **(B)** The classification accuracies for classification models based on sFC, dFC, and fusion of both metrics (sFC+dFC). DCC, dynamical conditional correlation; FLS, flexible least squares; AUC, area the under curve.

The classification accuracies were 65.9% and the corresponding AUCs of ROC curves were 0.66 for sFC-based predictive model, 79.6% and the corresponding AUCs of ROC curves were 0.88 for dFC-based predictive model (FLS approach), 65.9% and the corresponding AUCs of ROC curves were 0.73 for dFC-based predictive model (DCC approach), 81.8% and the corresponding AUCs of ROC curves were 0.92 for sFC fused dFC-based predictive model (FLS + sFC), 70.5% and the corresponding AUCs of ROC curves were 0.75 for sFC fused dFC-based predictive model (DCC + sFC) (Figures 7A, B). Supplementary Figure S3 illustrates the results of permutation tests.

### Validation analysis

To further perform internal validation of our current results, ICA was performed by adding 5 and subtracting 5 on the estimated number of components. The results from these validation analyses (e.g., 12-component level and 22-component level) were consistent with our main result (Supplementary Figures S4–S6, Supplementary Tables S3–S6). The SVM with the sigmoid kernel, using default parameters (C-SVC, penalty coefficient = 1, gamma = 0.1), produced the optimal model for this study (Supplementary Figures S7–S9). Furthermore, no difference in head motion was obtained between DCM patients and HC (Supplementary Figure S10).

## Discussion

In our study, three main findings emerged: (1) relative to HC, DCM patients exhibited significantly lower intra-network sFC and higher inter-network sFC between brain networks; (2) DCM patients exhibited a longer duration within a state characterized by heightened interconnectivity among brain networks, correlating with symptom duration; (3) Combining different FC metrics (such as sFC and dFC) improved classification accuracy between patients with good and poor recovery, indicating that integrating various FC measures offers a better neuroimaging marker for predicting DCM prognosis. These findings delineate abnormal coarse-grained intra- and inter-network connectivity in DCM patients and shed light on the underlying mechanisms of DCM.

Over the past decade, research has extensively examined fine-grained brain network abnormalities in DCM patients. Wang et al. examined holistic FC patterns across the entire brain using sFC analyses and found decreased connectivity within the sensorimotor network (SMN), correlating with spinal cord compression (3). They also observed compensatory increases in connectivity within and between primary and secondary sensorimotor regions, subcortical regions, visuospatial regions including the cuneus, as well as the brainstem and cerebellum (3). Similarly, Wei et al reported reduced FC strength within the primary motor cortex, indicating lower connections with other brain regions (22). Another structural study revealed significantly decreased fiber density (FD) and fiber cross-section (FDC) between DCM patients and HC along the corticospinal tract (23), encompassing regions spanning the corona radiate and internal capsule. By correlating FD and FDC with Neck Disability Index and modified JOA scores, they identified an augmentation in the total volume of projections to the thalamus, basal ganglia, and internal capsule. This was accompanied by heightened FC within the visual network (VN) and posterior parietal cortices. Cumulatively, these findings indicated a lower connectivity within the sensorimotor cortex associated with chronic spinal cord injury, with increased interactions among other brain networks to compensate for neurological impairments in DCM patients. Our results from intra-network connectivity analysis aligned with these prior discoveries. We identified significantly lower intra-network connectivity within SMN, VN, CBN, and DMN. These findings suggest that following the impairment of ascending and descending fiber tracts, information processing within specific brain regions has been altered, potentially reflecting the injured state of the compressed spinal cord.

Similarly to previous research, our inter-network connectivity analysis has identified compensatory changes in both static and dynamic FC. These compensatory changes in response to the gradual deterioration of spinal cord dysfunction serve to maintain sensorimotor function within a relatively normal range in DCM patients (24–27). This explanation for the higher FC in DCM patients is grounded in the mounting neuroimaging evidence suggesting that cortical reorganization through neuronal plasticity (28) occurs at both regional-level and network-level for compensating neurological deficits (3, 28, 29). fMRI studies have demonstrated that while the intensity of activations in motor cortices (such as the primary motor cortex and the supplementary motor area) was lower in DCM patients, the spatial extent of motor cortices activations expanded (i.e., Volume of activation), suggesting the recruitment of neighboring cortical regions in response to spinal cord impairment (30, 31). Additionally, fine-grained network analyses have revealed higher FC in certain circuits (e.g., thalamus-cortical circuit) (29), and functional networks (e.g., SMN, VN, and DMN) (3, 6, 8), indicating the compensatory reorganization across various brain levels. However, there is a noticeable absence of a holistic exploration of the coarse-grained inter-network interactions from a temporal perspective.

Our study revealed higher static and dynamic inter-network FC in DCM patients including the connectivity between AN and VN/CBN, and between CBN and aDMN which correlated with disease duration. Previous research by Bressler et al. has demonstrated that the nodes of the AN can influence activity in visual areas in a top-down manner (32, 33). The higher connectivity between LVN and AN in our study may indicate the brain allocates higher resources to refocus visual attention to environmental stimuli in DCM patients. Extensive cerebellar dysfunction has been reported in DCM patients (5, 34). Moreover, a previous study on corticocerebellar intrinsic functional connectivity indicated that the cerebellum is not a unitary structure (35). Specifically, Buckner et al. identified that cerebellar lobules VIIb and VIIIa exhibited connectivity with attention-related cortex, while cerebellar crus I and II couples with the cortical DMN. Taken together, in response to chronic spinal cord compression, coarse-grained inter-network reorganization occurs to compensate for neurological deficits in DCM patients.

Efforts to identify prognostic neuroimaging biomarkers for DCM have been ongoing. A previous study successfully predicted postoperative neurological recovery in DCM patients using static fine-grained brain functional network data, providing preliminary evidence for the use of fMRI data and machine learning techniques in predicting patient outcomes (17). The amplitude of low-frequency fluctuations in the primary motor cortex before surgery can provide additional information for predicting outcomes after decompression surgery, highlighting its potential utility as a prognostic biomarker for DCM patients (7). Furthermore, some studies have reported that preoperative ALFF in the left frontal pole and preoperative functional connectivity between the visual cortex and the right superior frontal gyrus could serve as biomarkers for postoperative recovery in DCM patients (36, 37). Implementing brain MRI analysis could benefit the prediction of outcomes after decompression surgery, but further work is still needed (38). Therefore, we conducted a machine learning analysis to assess the prognostic value of brain functional network abnormalities for patients.

In our study, using the same group of patients and classification algorithms, we achieved classification accuracies of 65.9% for sFC, 79.6% for dFC (FLS), and 65.9% for dFC (DCC). All these results are significantly higher than chance, indicating that different network measures contain valuable information for predicting the prognosis of patients.

Previous studies often focused on a single measure when distinguishing patients with different outcomes. Different measures capture network information from different aspects and thus provide complementary information. Combining information contained in different measures might enhance the classification performance. We found that combining dFC (FLS) and sFC improved accuracy by 2% (from 79.6% to 81.8%), while combining dFC (DCC) and sFC improved accuracy by 5% (from 65.9% to 70.5%). Fusion of measures yielded higher classification accuracy than using a single measure alone, suggesting that different network measures should be considered when developing a neuroimaging prognostic tool.

This study has a few limitations. First, the DCM patients in our study had received conservative treatment before surgery, which may have influenced our results to some extent. Second, the sample size may have restricted the accuracy of our study. Third, we didn't collect enough postoperative fMRI data due to the possible artifacts (e.g., plates and screws) and possible heating of these materials. Finally, more comprehensive demographic, clinical and behavioral assessments should be conducted in the future to thoroughly investigate brain reorganization in DCM patients by combining functional and structural MRI data.

## Conclusion

In conclusion, our findings provide valuable insights into the brain mechanisms underlying DCM neuropathology on the network level. DCM patients exhibit abnormal intra- and inter-network connectivity compared to HC. Combining static and dynamic FC can provide additional information for predicting the prognosis of DCM patients.

## Data Availability

The raw data supporting the conclusions of this article will be made available by the authors, without undue reservation.
