# Supplementary material for: Intra- and inter-network connectivity abnormalities associated with surgical outcomes in degenerative cervical myelopathy patients: a resting-state fMRI study
Source: Front Neurol. 2024 Nov 6;15:1490763. doi: 10.3389/fneur.2024.1490763 (PMC11580013; doi:10.3389/fneur.2024.1490763)
Supplement: Supplementary file 1 [file Data_Sheet_1.docx]

**Intra- and Inter-network Connectivity Abnormalities Associated with Surgical Outcomes in Degenerative Cervical Myelopathy Patients: A Resting-State fMRI Study**

**Table S1.** Peak activation information of identified 11 independent components

| **Intrinsic connectivity network** | | **BA** | **Voxels** | ***T*-value** | **Peak MNI** | | |
| --- | --- | --- | --- | --- | --- | --- | --- |
|  |  |  |  |  | **x** | **y** | **z** |
| **Medial visual network (MVN)** | |  |  |  |  |  |  |
| IC 1 | Bi calcarine gyrus | 17,18 | 3429 | 28.00 | 6 | -78 | 9 |
| **Lateral visual network (LVN)** | |  |  |  |  |  |  |
| IC 3 | R fusiform gyrus | 19,37 | 1517 | 24.60 | 33 | -81 | 21 |
|  | L fusiform gyrus | 19,37 | 1281 | 21.21 | -33 | -87 | 12 |
| **Cerebellar network (CBN)** | |  |  |  |  |  |  |
| IC 2 | Bi cerebellum | — | 1692 | 20.34 | -6 | -81 | -27 |
| **Anterior default mode network (aDMN)** | |  |  |  |  |  |  |
| IC 4 | Bi middle frontal gyrus | 8,32 | 4815 | 23.24 | 24 | 45 | 30 |
| **Posterior default mode network (pDMN)** | |  |  |  |  |  |  |
| IC 6 | Bi posterior cingulate | 23 | 3476 | 24.31 | -3 | -72 | 30 |
| **Attention network (AN)** | |  |  |  |  |  |  |
| IC 7 | R superior temporal gyrus | 20,21 | 2184 | 20.04 | 54 | -57 | 12 |
|  | L superior temporal gyrus | 20,21 | 1291 | 22.47 | -51 | -63 | 15 |
|  | L inferior frontal gyrus | 44,48 | 661 | 17.38 | -54 | 15 | 3 |
|  | Bi Cingulate Gyrus | 32 | 792 | 16.04 | -3 | 9 | 57 |
|  | Bi precuneus | 7 | 178 | 11.69 | -6 | -57 | 36 |
| **Auditory network (AUN)** | |  |  |  |  |  |  |
| IC 8 | R superior temporal gyrus | 22 | 1933 | 20.99 | 60 | -9 | 3 |
|  | L superior temporal gyrus | 22 | 972 | 17.14 | -57 | -21 | 12 |
| **Left frontoparietal network (LFPN)** | |  |  |  |  |  |  |
| IC 13 | L inferior frontal gyrus**/**  inferior parietal lobule | 40,48 | 3901 | 27.18 | -45 | 24 | 18 |
|  | Bi medial frontal gyrus | 32 | 290 | 15.52 | -3 | 12 | 54 |
| **Right frontoparietal network (RFPN)** | |  |  |  |  |  |  |
| IC 14 | R middle frontal gyrus | 44,45 | 1797 | 18.35 | 42 | 33 | 36 |
|  | R inferior parietal lobule | 40 | 1130 | 29.92 | 57 | -51 | 36 |
|  | L inferior parietal lobule | 40 | 540 | 20.17 | -54 | 51 | 45 |
| **Motor network (MN)** | |  |  |  |  |  |  |
| IC 16 | Bi Precentral Gyrus | 6 | 7104 | 20.83 | 57 | -6 | 30 |
| **Sensory network (SN)** | |  |  |  |  |  |  |
| IC 12 | Bi postcentral gyrus | 3 | 4841 | 24.60 | 45 | -36 | 51 |

IC, independent component; MNI, Montreal Neurologic Institute; BA, Brodmann area; R, right; L, left; Bi, bilateral.

**Table S2.** The DSC between the template and spatial maps of each component

| **RSN** | **Dice similarity coefficient** |
| --- | --- |
| MVN | 0.76 |
| LVN | 0.53 |
| CBN | 0.64 |
| aDMN | 0.54 |
| pDMN | 0.53 |
| AN | 0.55 |
| AUN | 0.65 |
| LFPN | 0.52 |
| RFPN | 0.54 |
| SN | 0.57 |
| MN | 0.67 |

DSC, dice similarity coefficient; RSN, resting-state network; MVN, medial visual network; LVN, lateral visual network; CBN, cerebellar network; aDMN, anterior default mode network; pDMN, posterior default mode network; AN, attention network; AUN, auditory network; LFPN, left frontoparietal network; RFPN, right frontoparietal network; SN, sensory network; MN, motor network.

**Table S3**. Peak activation information of identified 9 independent components at 12-component level

| **Intrinsic connectivity network** | | **BA** | **Voxels** | ***T*-value** | **Peak MNI** | | |
| --- | --- | --- | --- | --- | --- | --- | --- |
|  |  |  |  |  | **x** | **y** | **z** |
| **Visual network (VN)** | |  |  |  |  |  |  |
| IC 2 | Bi calcarine gyrus/ lingual gyrus | 17,18 | 4527 | 28.20 | 3 | -75 | 12 |
| **Cerebellar network (CBN)** | |  |  |  |  |  |  |
| IC 4 | Bi cerebellum posterior lobe | — | 1514 | 19.65 | 3 | -48 | -9 |
| **Anterior default mode network (aDMN)** | |  |  |  |  |  |  |
| IC 3 | Bi middle frontal gyrus | 8,32 | 5207 | 21.46 | 24 | 48 | 27 |
| **Posterior default mode network (pDMN)** | |  |  |  |  |  |  |
| IC 12 | Bi Posterior Cingulate | 23,30 | 1445 | 26.10 | -3 | -54 | 24 |
|  | L middle temporal gyrus | 39 | 432 | 19.90 | -33 | -69 | 36 |
|  | R middle temporal gyrus | 39 | 369 | 20.13 | 48 | -66 | 27 |
| **Attention network (AN)** | |  |  |  |  |  |  |
| IC 9 | L superior temporal gyrus | 20,21 | 2816 | 24.11 | -54 | -60 | 27 |
|  | R superior temporal gyrus | 20,21 | 1296 | 19.02 | 60 | -57 | 27 |
|  | Bi superior frontal gyrus/  cingulate gyrus | 32 | 1076 | 16.23 | -3 | 21 | 57 |
|  | Bi Precuneus | 23 | 547 | 17.39 | -9 | -57 | 33 |
|  | L middle frontal gyrus | 9 | 247 | 14.05 | -42 | 6 | 48 |
| **Auditory network (AUN)** | |  |  |  |  |  |  |
| IC 8 | R superior temporal gyrus | 22 | 2479 | 20.83 | 57 | 0 | -12 |
|  | L superior temporal gyrus | 22 | 817 | 15.33 | -66 | -24 | 15 |
| **Left frontoparietal network (LFPN)** | |  |  |  |  |  |  |
| IC 7 | L middle frontal gyrus | 44,45 | 2224 | 23.16 | -45 | 30 | 18 |
|  | L inferior parietal lobule | 40 | 1218 | 22.51 | -33 | 54 | 42 |
| **Right frontoparietal network (RFPN)** | |  |  |  |  |  |  |
| IC 6 | R middle frontal gyrus | 44,46 | 1106 | 15.18 | 42 | 15 | 48 |
|  | R inferior parietal lobule | 40 | 765 | 32.07 | 48 | -54 | 45 |
|  | L inferior parietal lobule | 40 | 696 | 21.92 | -54 | -51 | 45 |
| **Sensorimotor network (SMN)** | |  |  |  |  |  |  |
| IC 1 | Bi precentral gyrus/  postcentral gyrus/  supramarginal gyrus | 2,3,6 | 9665 | 25.839 | 30 | -39 | 60 |

IC, independent component; MNI, Montreal Neurologic Institute; BA, Brodmann area; R, right; L, left; Bi, bilateral.

**Table S4.** Peak activation information of identified 11 independent components at 22-component level

| **Intrinsic connectivity network** | | **BA** | **Voxels** | ***T*-value** | **Peak MNI** | | |
| --- | --- | --- | --- | --- | --- | --- | --- |
|  |  |  |  |  | **x** | **y** | **z** |
| **Medial visual network (MVN)** | |  |  |  |  |  |  |
| IC 2 | Bi calcarine gyrus/ lingual gyrus | 17,18 | 3386 | 28.49 | -3 | -72 | 12 |
| **Lateral visual network (LVN)** | |  |  |  |  |  |  |
| IC 5 | R fusiform gyrus | 19,37 | 1468 | 23.33 | 33 | -81 | 21 |
|  | L fusiform gyrus | 19,37 | 1474 | 22.73 | -24 | -75 | 30 |
| **Cerebellar network (CBN)** | |  |  |  |  |  |  |
| IC 7 | Bi cerebellum posterior lobe | — | 1900 | 18.97 | 3 | -48 | -9 |
| **Anterior default mode network (aDMN)** | |  |  |  |  |  |  |
| IC 11 | R middle frontal gyrus | 8,32 | 4429 | 24.40 | 33 | 54 | 15 |
|  | Bi precuneus | 7 | 85 | 11.57 | -6 | 60 | 54 |
| **Posterior default mode network (pDMN)** | |  |  |  |  |  |  |
| IC 3 | Bi posterior cingulate | 23 | 1845 | 23.07 | 0 | -72 | 36 |
|  | R angular gyrus | 40 | 137 | 11.63 | 36 | -60 | 39 |
|  | L angular gyrus | 40 | 137 | 11.97 | -33 | -54 | 42 |
| **Attention network (AN)** | |  |  |  |  |  |  |
| IC 21 | R superior temporal gyrus | 20,21 | 2106 | 22.22 | 54 | -51 | 15 |
|  | L superior temporal gyrus | 20,21 | 1462 | 23.10 | -54 | -57 | 21 |
|  | Bi superior frontal gyrus | 8 | 537 | 15.24 | 6 | 15 | 60 |
|  | Bi precuneus | 7 | 496 | 15.84 | 6 | -54 | 42 |
|  | L inferior frontal gyrus | 48 | 381 | 14.02 | 54 | 18 | 9 |
| **Auditory network (AUN)** | |  |  |  |  |  |  |
| IC 14 | R superior temporal gyrus | 22 | 1787 | 24.27 | 57 | -9 | 0 |
|  | L superior temporal gyrus | 22 | 830 | 16.99 | -60 | -12 | 6 |
| **Left frontoparietal network (LFPN)** | |  |  |  |  |  |  |
| IC 18 | L middle frontal gyrus | 44,45 | 1858 | 15.20 | -30 | 9 | 57 |
|  | L inferior parietal lobule | 40 | 1279 | 22.25 | -54 | -57 | 42 |
| **Right frontoparietal network (RFPN)** | |  |  |  |  |  |  |
|  | R middle frontal gyrus | 32,45 | 1217 | 17.70 | 42 | 33 | 36 |
|  | R inferior parietal lobule | 40 | 1031 | 29.10 | 57 | -51 | 36 |
|  | L inferior parietal lobule | 40 | 449 | 20.75 | -54 | -51 | 45 |
| **Motor network (MN)** | |  |  |  |  |  |  |
| IC 4 | R precentral gyrus | 6 | 999 | 22.03 | 57 | -6 | 30 |
|  | L precentral gyrus | 6 | 1105 | 22.87 | 60 | -12 | 36 |
| **Sensory network (SN)** | |  |  |  |  |  |  |
| IC 6 | Bi postcentral gyrus | 2,3 | 5233 | 25.05 | -21 | -39 | 63 |

IC, independent component; MNI, Montreal Neurologic Institute; BA, Brodmann area; R, right; L, left; Bi, bilateral.

**Table S5.** Abnormal functional connectivity in each RSN between DCM patients and HC at 12-component level

| **RSN** | **Regions** | **BA** | **Voxels** | ***T*-value** | **Peak MNI** | | |
| --- | --- | --- | --- | --- | --- | --- | --- |
|  |  |  |  |  | **x** | **y** | **z** |
| **DCM < HC** | | | | | | | |
| VN | R lingual gyrus | 18,19 | 46 | -5.09 | 24 | -54 | -3 |
| CBN | R cerebellum posterior lobe | — | 291 | -6.34 | 21 | -75 | -30 |
|  | L cerebellum posterior lobe | — | 117 | -6.27 | -30 | -72 | -30 |
| pDMN | Bi precuneus | 23 | 31 | -4.51 | 0 | -66 | 27 |
| AN | L middle temporal gyrus | 20 | 107 | -5.97 | -57 | -9 | -24 |
|  | R superior temporal gyrus | 21 | 35 | -5.69 | 51 | 6 | -27 |
|  | R middle temporal gyrus | 21 | 33 | -5.76 | 63 | -6 | -18 |
| SMN | L precentral gyrus | 4,6 | 72 | -5.16 | -45 | -6 | 27 |
|  | L precentral gyrus | 4,6 | 46 | -5.02 | -54 | -9 | 45 |

DCM, degenerative cervical myelopathy; HC, healthy controls; IC, independent component; MNI, Montreal Neurologic Institute; BA, Brodmann area; RSN, resting-state network; VN, visual network; CBN, cerebellar network; pDMN, posterior default mode network; AN, attention network; SMN, sensorimotor network, R, right; L, left; Bi, bilateral.

**Table S6.** Abnormal functional connectivity in each RSN between DCM patients and HC at 22-component level

| **RSN** | **Regions** | **BA** | **Voxels** | ***T*-value** | **Peak MNI** | | |
| --- | --- | --- | --- | --- | --- | --- | --- |
|  |  |  |  |  | **x** | **y** | **z** |
| **DCM < HC** | | | | | | | |
| MVN | R lingual gyrus | 18 | 91 | -5.52 | 21 | -54 | -3 |
| CBN | Bi cerebellum posterior lobe | — | 642 | -5.93 | 36 | -69 | -30 |
| pDMN | Bi precuneus | 23 | 68 | 4.5543 | -6 | -75 | 27 |
| AN | L middle temporal gyrus | 21 | 31 | -4.61 | -54 | -9 | -12 |
| MN | L precentral gyrus | 6 | 93 | -5.60 | -54 | -3 | 30 |
|  | R precentral gyrus | 6 | 76 | -6.06 | 54 | -3 | 27 |

DCM, degenerative cervical myelopathy; HC, healthy controls; IC, independent component; MNI, Montreal Neurologic Institute; BA, Brodmann area; RSN, resting-state network; MVN, medial visual network; CBN, cerebellar network; pDMN, posterior default mode network; AN, attention network; MN, motor network; R, right; L, left; Bi, bilateral.

**Figure S1**


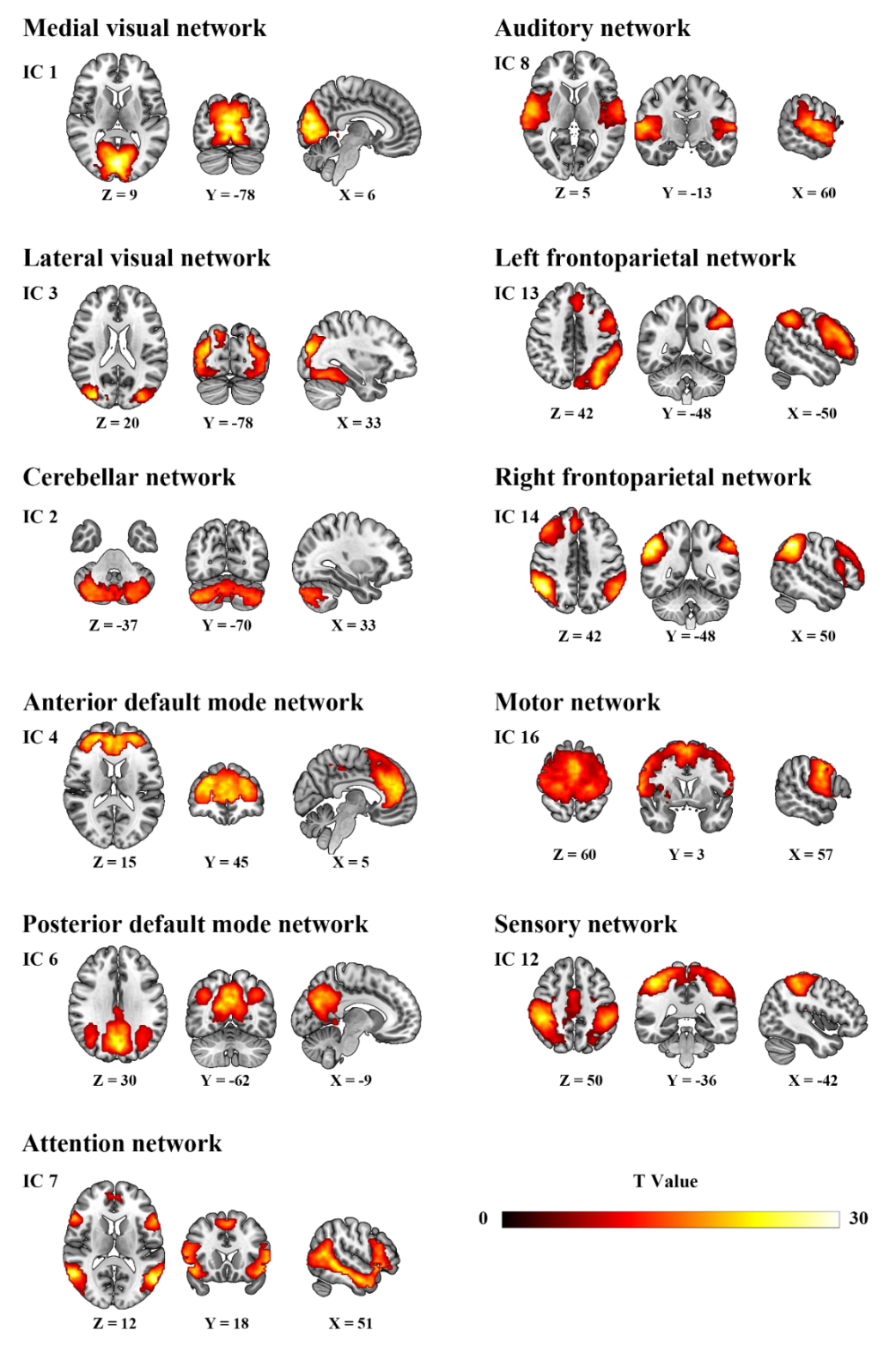


Eleven brain networks identified in independent component analysis. IC, independent component.

**Figure S2**


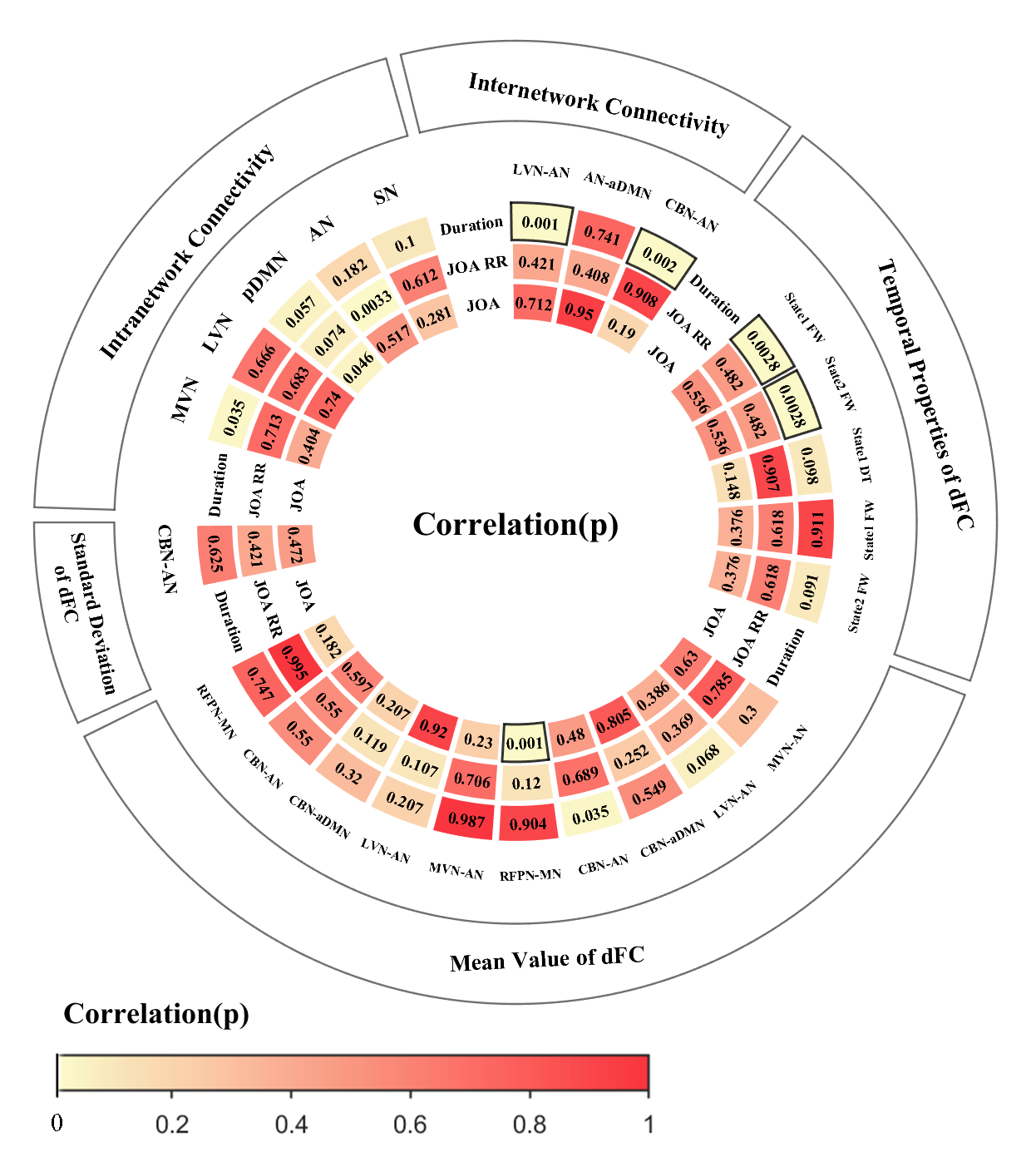


The results of P-value before FDR correction in the correlation analysis. sFC, static functional connectivity; dFC, dynamic functional connectivity; DCC, dynamical conditional correlation; FLS, flexible least squares; S1, state1; S2, state2; FT, fraction time; MDT, mean dwell time; JOA, Japanese Orthopedic Association; JOA RR, JOA recovery rate; LVN, lateral visual network; AN, attention network; CBN, cerebellar network; RFPN, right frontoparietal network; MN, motor network; MVN, medial visual network; aDMN, anterior default mode network; pDMN, posterior default mode network; AUN, auditory network; LFPN, left frontoparietal network; SN, sensory network.

**Figure S3**


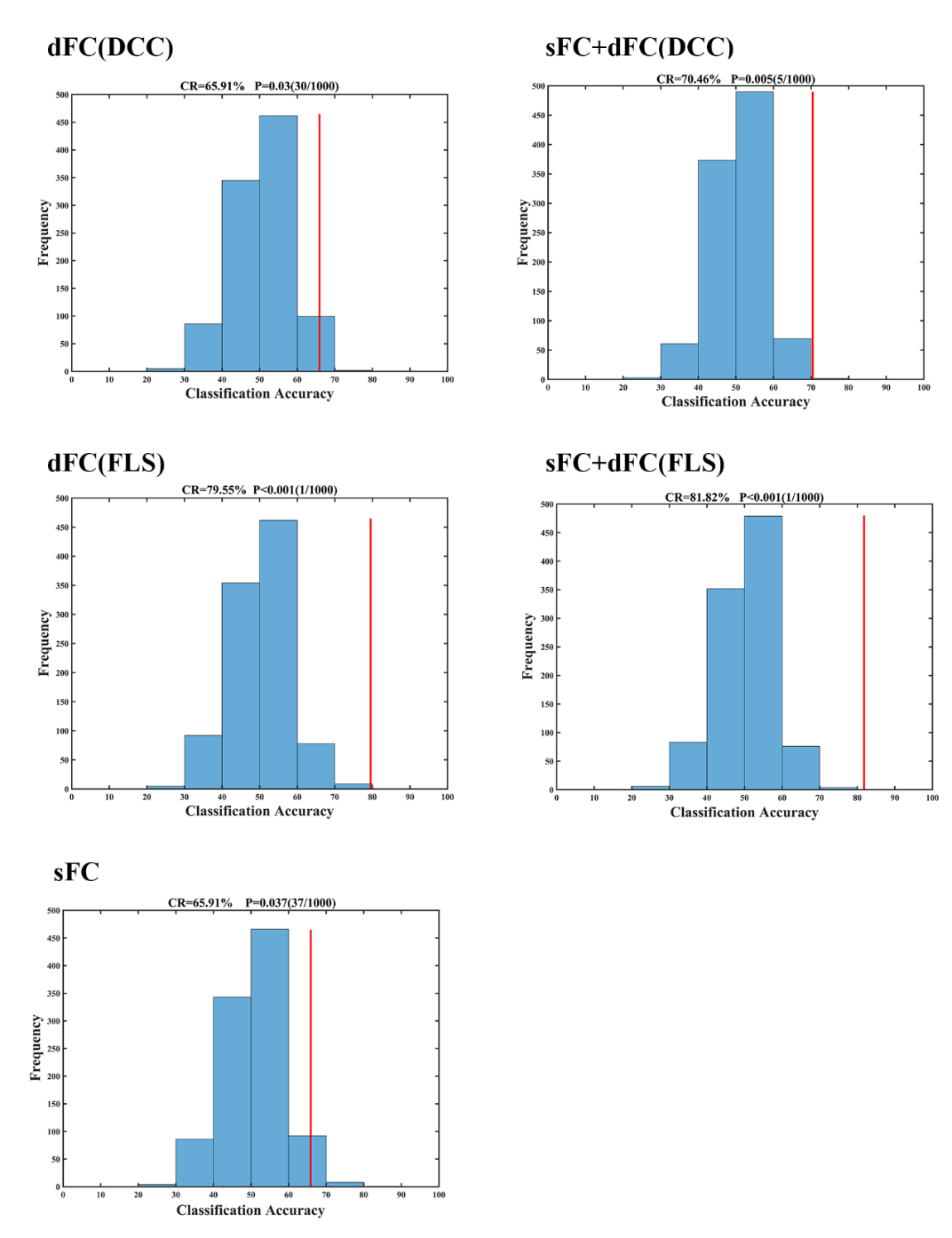


The results of permutation tests in multi-variate pattern analysis. The result of permutation test for sFC-based predictive model, dFC-based predictive model (DCC approach), dFC-based predictive model (FLS approach), sFC fused dFC-based predictive model (DCC approach + sFC), and sFC fused dFC-based predictive model (FLS approach + sFC). sFC, static functional connectivity; dFC, dynamic functional connectivity; DCC, dynamical conditional correlation; FLS, flexible least squares; CR, correct rate.

**Figure S4**


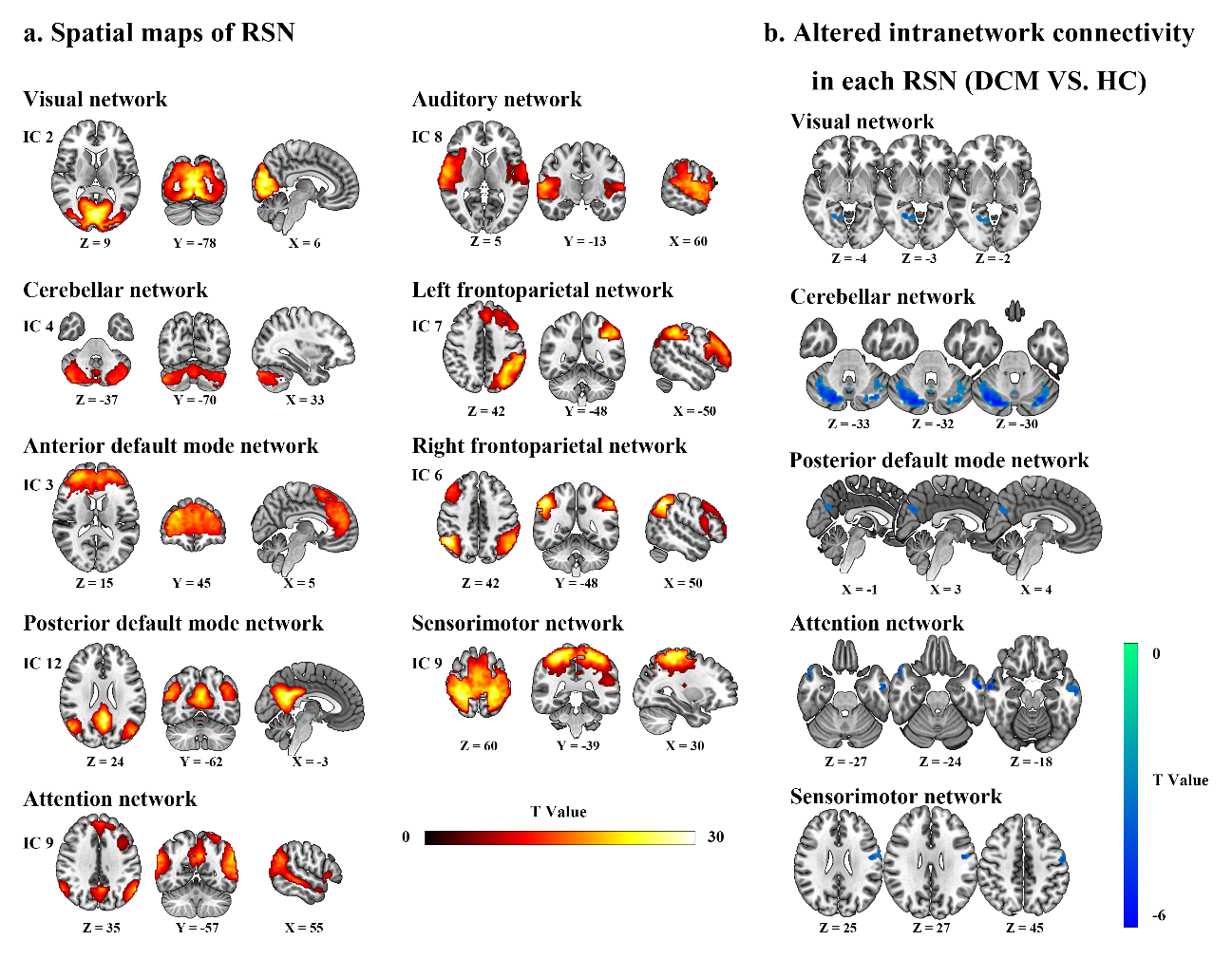


Panel (a): Nine brain networks identified in independent component analysis at 12-component level. Panel (b): Brain regions with significant differences between degenerative cervical myelopathy patients (DCM) and healthy controls (HC) within each resting-state network (RSN) at 12-component level. IC, independent component.

**Figure S5**


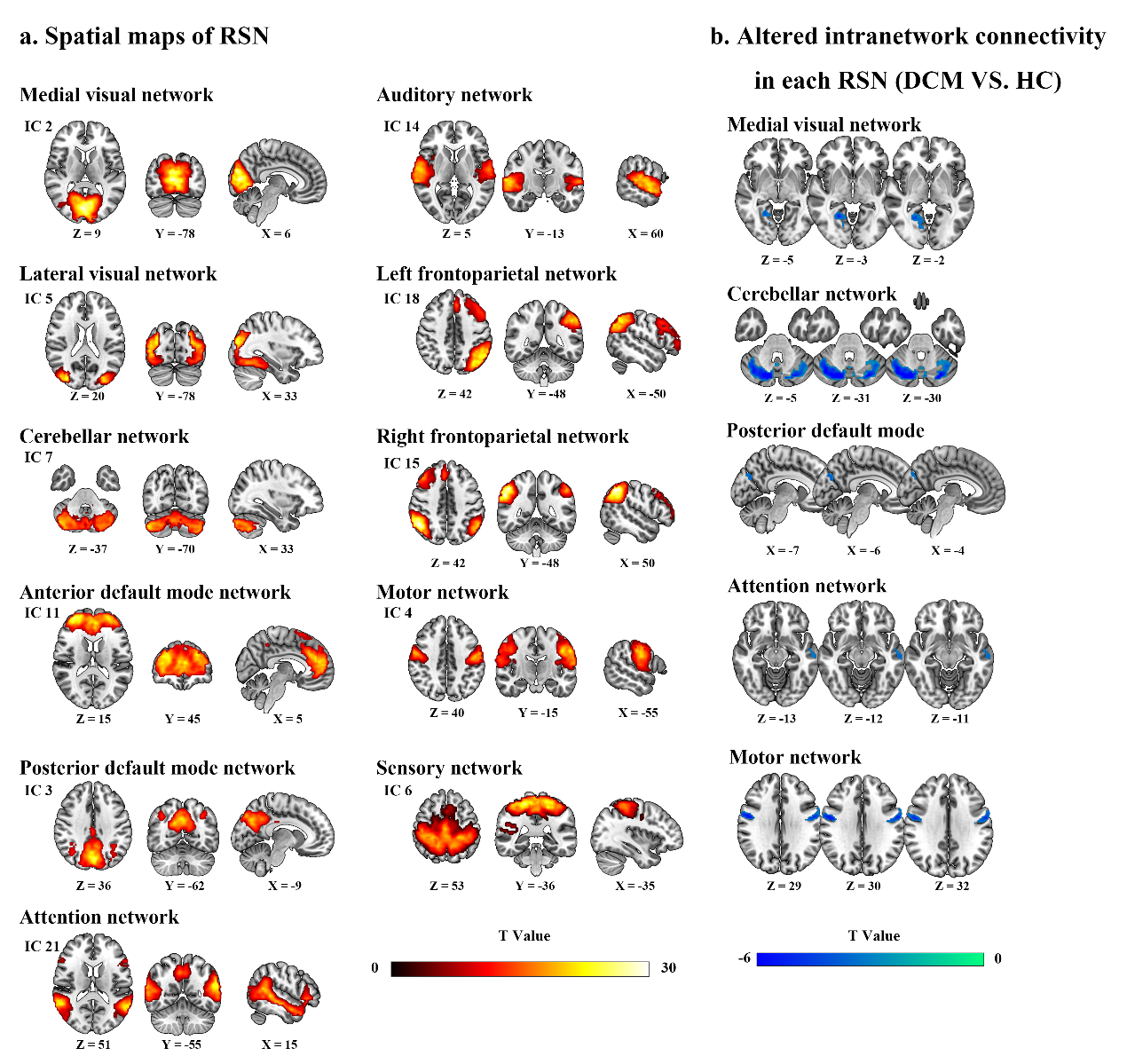


Panel (a): Eleven brain networks identified in independent component analysis at 22-component level. Panel (b): Brain regions with significant differences between degenerative cervical myelopathy patients (DCM) and healthy controls (HC) within each resting-state network (RSN) at 22-component level. IC, independent component.

**Figure S6**


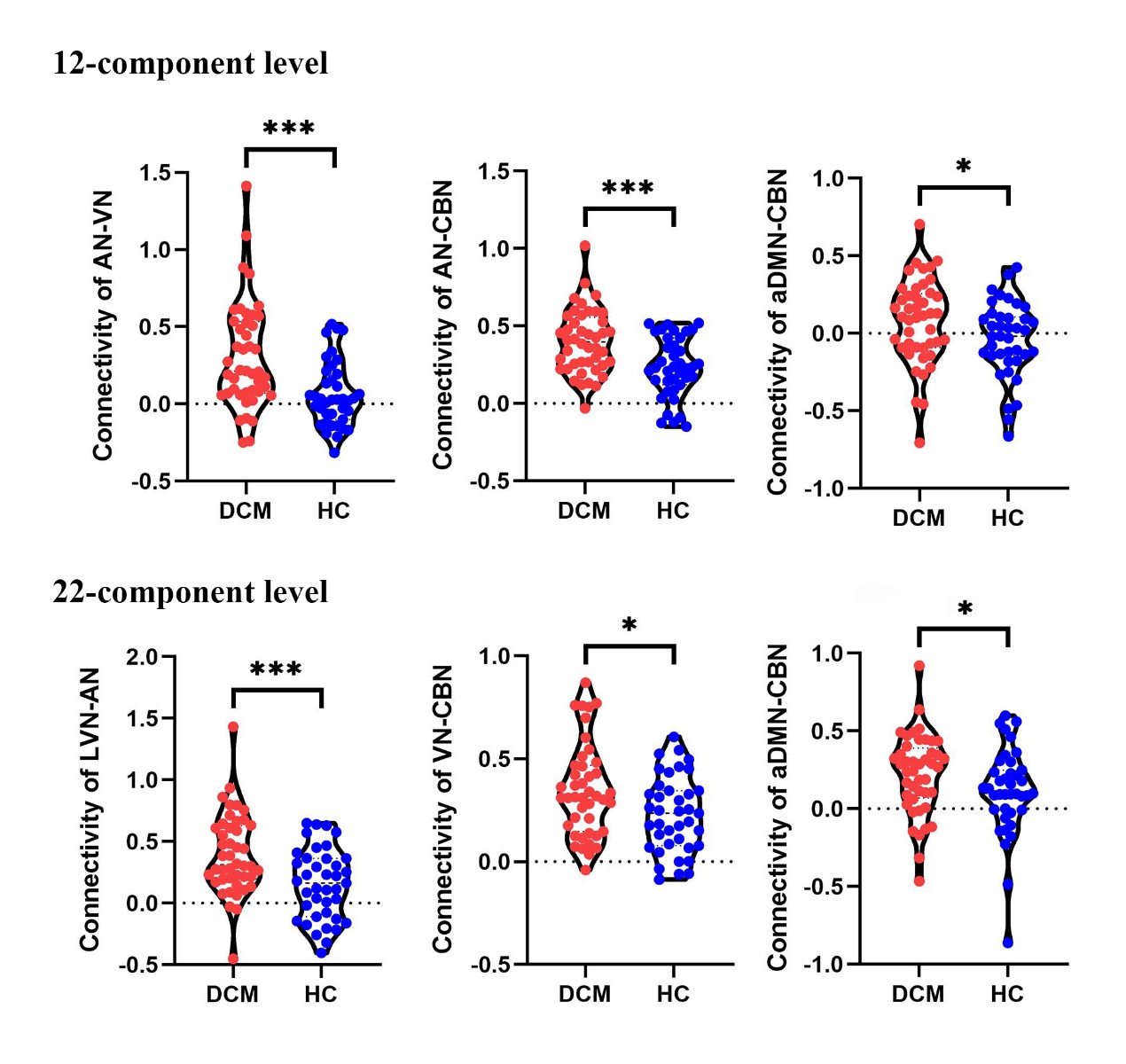


The between-group differences in internetwork connectivity between AN and VN, between AN and CBN, and between aDMN and CBN at 12-component level and at 22-component level respectively. DCM, degenerative cervical myelopathy; HC, healthy controls; AN, attention network; VN, visual network; LVN, lateral visual network; CBN, cerebellar network; aDMN, anterior default mode network.

**Figure S7**


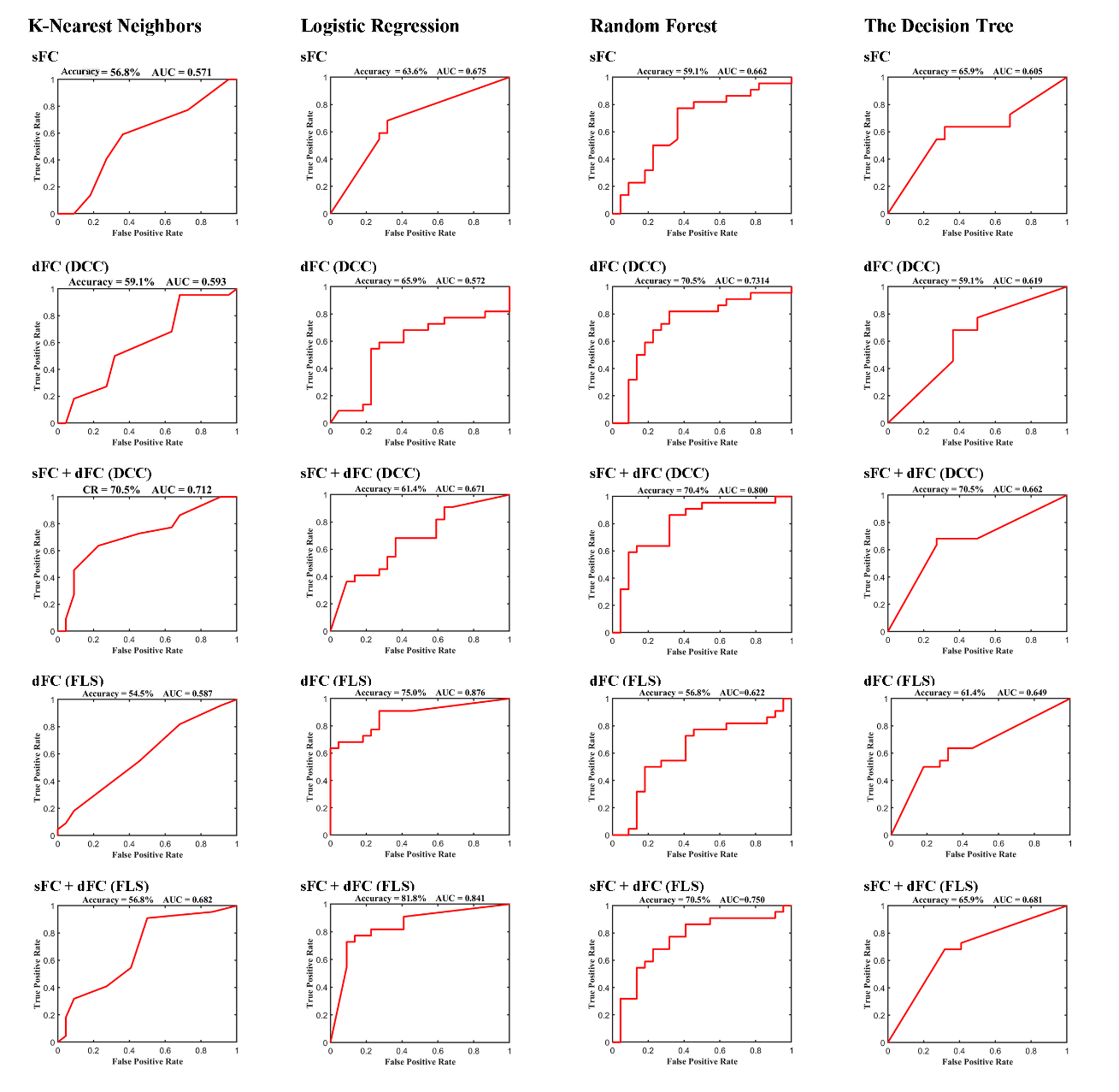


The results of validation analysis using different algorithms with default parameters to construct predictive models. DCC, dynamical conditional correlation; FLS, flexible least squares; sFC, static functional connectivity; dFC, dynamic functional connectivity; AUC, area the under curve.

**Figure S8**


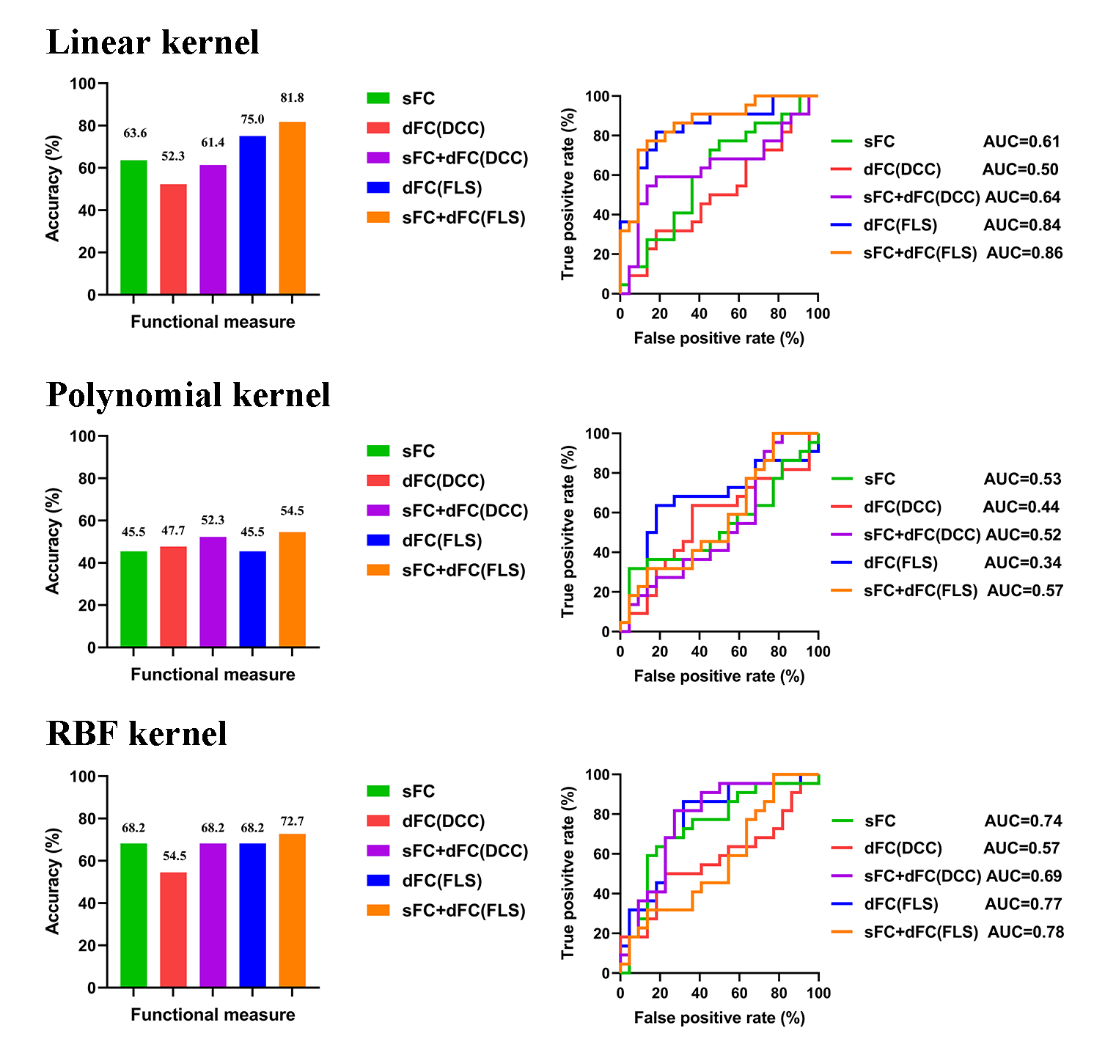


The results of validation analysis using different kernel of support vector machine with default parameters to construct predictive models. DCC, dynamical conditional correlation; FLS, flexible least squares; sFC, static functional connectivity; dFC, dynamic functional connectivity; AUC, area the under curve.

**Figure S9**


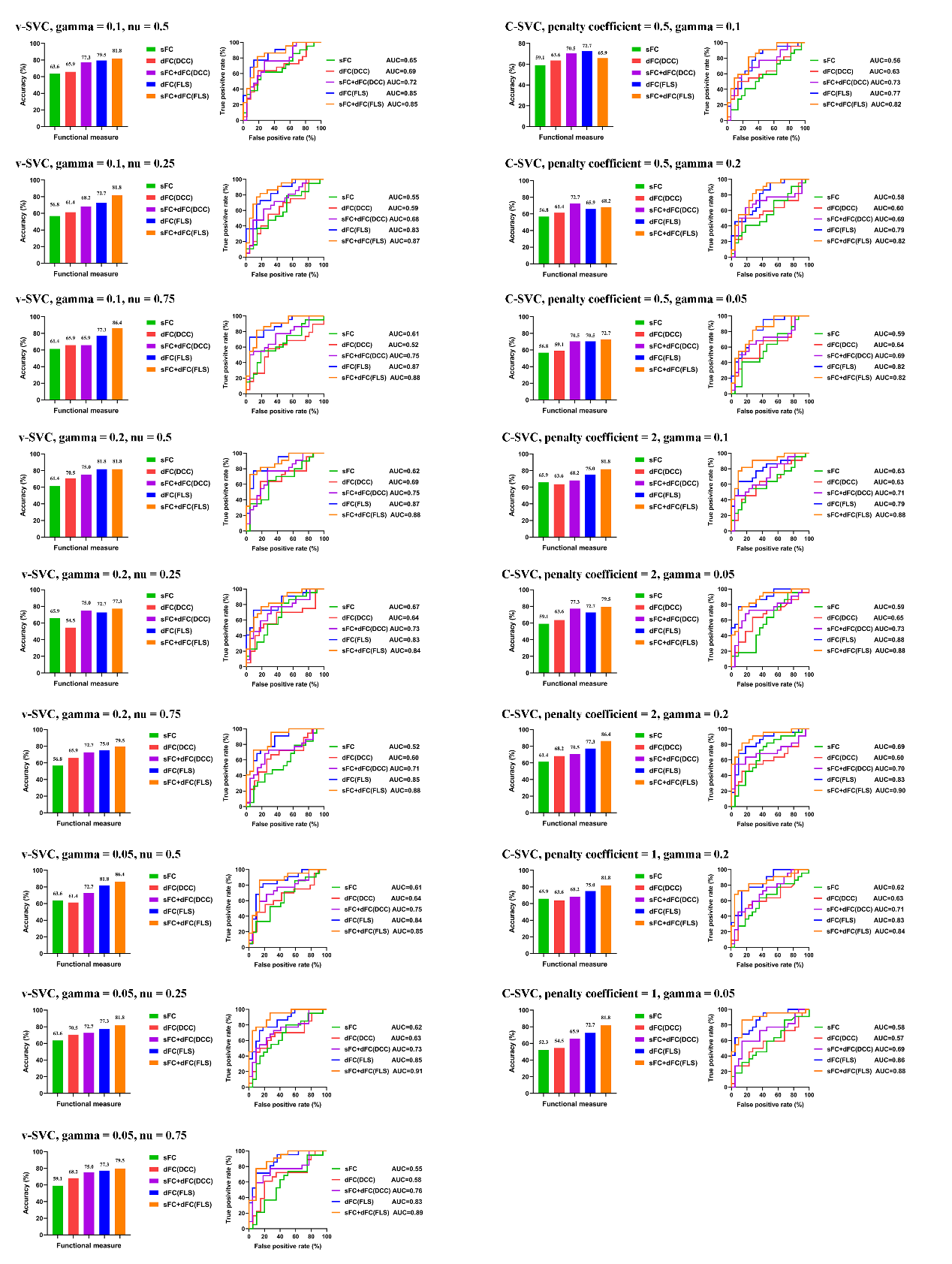


The results of validation analysis using different parameters of support vector machine with sigmoid kernel to construct predictive models. DCC, dynamical conditional correlation; FLS, flexible least squares; sFC, static functional connectivity; dFC, dynamic functional connectivity; AUC, area the under curve.

**Figure S10**


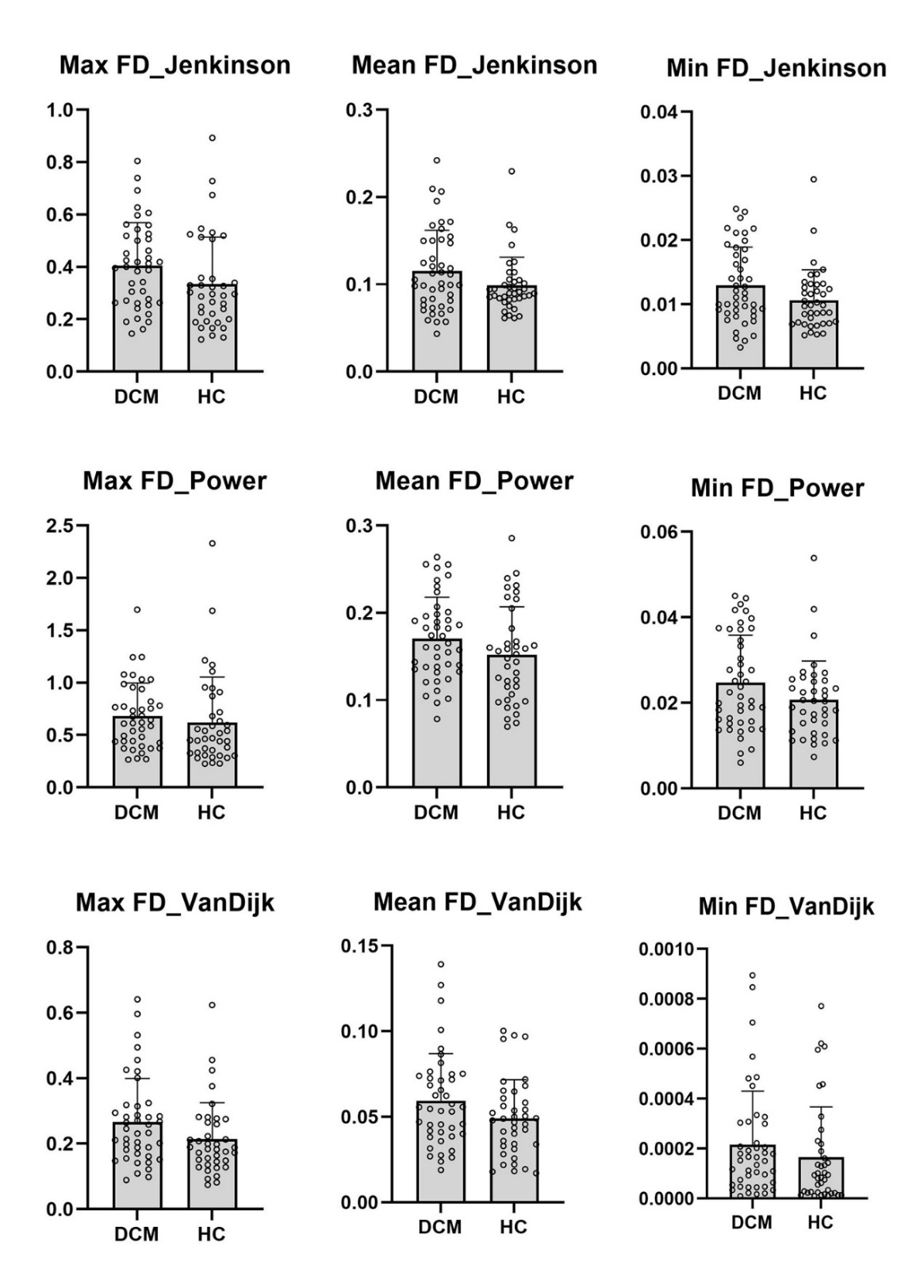


Min, mean and max framewise displacement (FD) value (i.e., FD Jenkinson, FD Power, FD VanDijk) differences between degenerative cervical myelopathy (DCM) patients and healthy controls (HC).
